# Identification of flubendazole as potential anti-neuroblastoma compound in a large cell line screen

**DOI:** 10.1038/srep08202

**Published:** 2015-02-03

**Authors:** Martin Michaelis, Bishr Agha, Florian Rothweiler, Nadine Löschmann, Yvonne Voges, Michel Mittelbronn, Tatjana Starzetz, Patrick N. Harter, Behnaz A. Abhari, Simone Fulda, Frank Westermann, Kristoffer Riecken, Silvia Spek, Klaus Langer, Michael Wiese, Wilhelm G. Dirks, Richard Zehner, Jaroslav Cinatl, Mark N. Wass, Jindrich Cinatl

**Affiliations:** 1 Institut für Medizinische Virologie, Klinikum der Goethe-Universität, Paul Ehrlich-Str. 40, 60596 Frankfurt am Main, Germany; 2 Centre for Molecular Processing and School of Biosciences, University of Kent, Canterbury CT2 7NJ, UK; 3 Neurological Institute (Edinger Institute), Goethe University, Heinrich-Hoffmann Strasse 7, 60528 Frankfurt am Main, Germany; 4 Institut für Experimentelle Tumorforschung in der Pädiatrie, Klinikum der Goethe-Universität, Komturstrasse 3a, 60528 Frankfurt am Main, Germany; 5 Division Tumor Genetics, B030, German Cancer Research Center, Im Neuenheimer Feld 280, 69120 Heidelberg, Germany; 6 Forschungsabteilung Zell- und Gentherapie, Interdisziplinäre Klinik und Poliklinik für Stammzelltransplantation, Universitätsklinikum Hamburg-Eppendorf, Martinistrasse 52, 20246 Hamburg, Germany; 7 Institut für Pharmazeutische Technologie und Biopharmazie, Westfälische Wilhelms-Universität Münster, Corrensstrasse 48, 48149 Münster, Germany; 8 Pharmaceutical Institute, University of Bonn, An der Immenburg 4, 53121 Bonn, Germany; 9 Leibniz-Institute Deutsche Sammlung für Mikroorganismen und Zellkulturen GmbH, Inhoffenstraße 7B, 38124 Braunschweig, Germany; 10 Institut für Rechtsmedizin, Klinikum der Goethe-Universität, Kennedyallee 104, 60596 Frankfurt am Main, Germany

## Abstract

Flubendazole was shown to exert anti-leukaemia and anti-myeloma activity through inhibition of microtubule function. Here, flubendazole was tested for its effects on the viability of in total 461 cancer cell lines. Neuroblastoma was identified as highly flubendazole-sensitive cancer entity in a screen of 321 cell lines from 26 cancer entities. Flubendazole also reduced the viability of five primary neuroblastoma samples in nanomolar concentrations thought to be achievable in humans and inhibited vessel formation and neuroblastoma tumour growth in the chick chorioallantoic membrane assay. Resistance acquisition is a major problem in high-risk neuroblastoma. 119 cell lines from a panel of 140 neuroblastoma cell lines with acquired resistance to various anti-cancer drugs were sensitive to flubendazole in nanomolar concentrations. Tubulin-binding agent-resistant cell lines displayed the highest flubendazole IC_50_ and IC_90_ values but differences between drug classes did not reach statistical significance. Flubendazole induced p53-mediated apoptosis. The siRNA-mediated depletion of the p53 targets p21, BAX, or PUMA reduced the neuroblastoma cell sensitivity to flubendazole with PUMA depletion resulting in the most pronounced effects. The MDM2 inhibitor and p53 activator nutlin-3 increased flubendazole efficacy while RNAi-mediated p53-depletion reduced its activity. In conclusion, flubendazole represents a potential treatment option for neuroblastoma including therapy-refractory cells.

Anthelmintic benzimidazoles including mebendazole and flubendazole were shown to exert anti-cancer activity by mechanisms including inhibition of microtubule function^1^,^2^,^3^,^4^,^5^,^6^,^7^.

Mebendazole affects the viability of cancer cells in experimental systems from a broad spectrum of cancer entities including lung cancer, breast cancer, ovary cancer, adrenocortical carcinoma, osteosarcoma, melanoma, glioblastoma, and colorectal carcinoma^1^,^3^,^4^,^5^,^6^,^7^. More recently, flubendazole was shown to affect the viability of leukaemia and myeloma cells in nanomolar concentrations^2^.

Here, we performed a screen of flubendazole in a panel of 321 cell lines including cell lines from 26 cancer entities. Among leukaemia and multiple myeloma, neuroblastoma was identified as a highly flubendazole-sensitive cancer entity. Flubendazole showed broad activity in primary neuroblastoma cells and a panel of 140 neuroblastoma cell lines with acquired drug resistance. The anti-neuroblastoma activity of flubendazole involved p53-mediated apoptosis and the MDM2 inhibitor and p53 activator nutlin-3 strongly enhanced the flubendazole effects. A water-soluble flubendazole-(2-hydroxypropyl)-β-cyclodextrin preparation inhibited vessel formation and tumour growth in the chick chorioallantoic membrane (CAM) model in vivo.

## Results

### Effects of flubendazole on cancer cell viability in a panel of 321 cancer cell lines from 26 cancer entities

**Figure 1:**
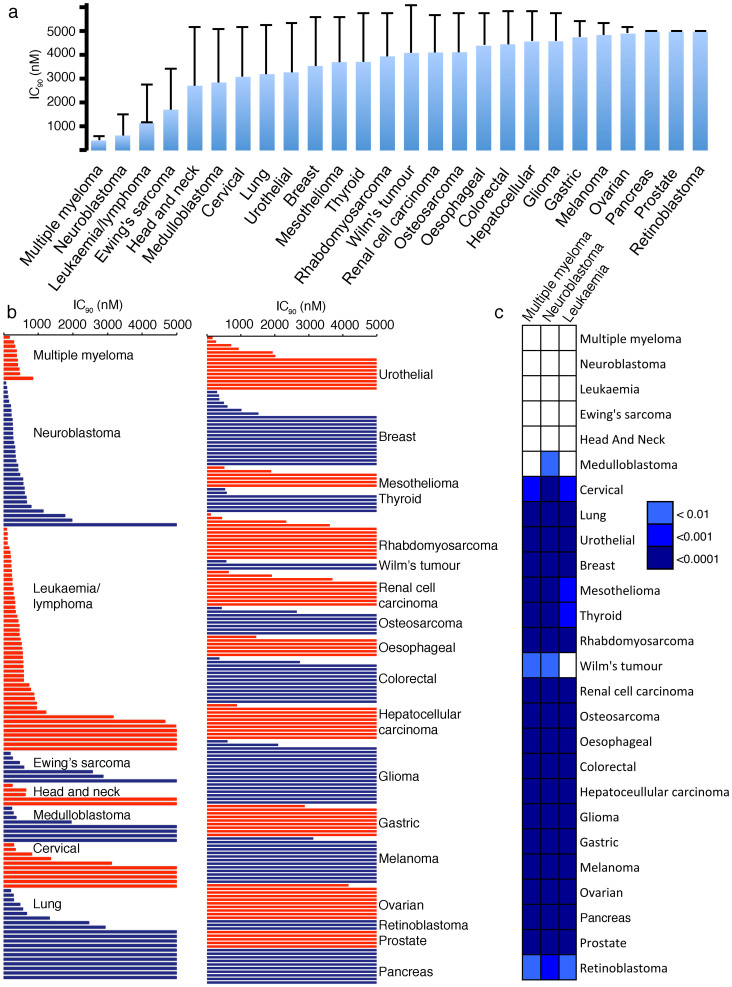
Effects of flubendazole in a panel of cancer cell lines from different entities. The flubendazole concentrations that decreased cancer cell viability by 90% (IC_90_) were determined by MTT assay after 5 days of incubation (flubendazole 5 μM was the maximum concentration tested). (a) The average IC_90_ values for each cancer entity are presented. Error bars represent a single standard deviation; (b) The average IC_90_ for each cell line (average from 2 experiments per cell line) is presented. Cancer entities are ordered by increasing overall average as shown in A. (c) The Wilcoxon rank sum test indicated significant differences (p < 0.05) between IC_90_s for the different cancer entities. Cells are coloured according to their significance level after multiple testing correction applying the Benjamini-Hochberg method. The detailed data is shown in Suppl. Table 1.

Flubendazole was screened in a panel of 321 cancer cell lines from 26 cancer entities. Concentrations of 1 μM appear pharmacologically achievable based on prior studies in mice that demonstrated a dose of 5 mg/kg to produce a C_max_ of 3.6 μM^8^. The maximum concentration tested was 5 μM. Multiple myeloma, neuroblastoma, and leukaemia/lymphoma consistently belonged to the cancer entities that displayed the highest sensitivity to flubendazole (Fig. 1a, Suppl. Fig. S1, Suppl. Table S1). This confirmed previous investigations that had suggested multiple myeloma and leukaemia to be flubendazole-sensitive cancer types^2^ and identified neuroblastoma as an additional flubendazole-sensitive entity. Statistical testing using the Wilcoxon rank sum test^9^ with subsequent Benjamini-Hochberg correction^10^ indicated the flubendazole IC_90_ values in neuroblastoma cells to be significantly lower than those from 21 out of the 25 other investigated cancer cell types (Fig. 1c).

117 (36%) of the 321 cell lines displayed IC_90_s < 1 μM. 31 cell lines (10%) displayed IC_90_s > 1 μM and <5 μM, and 173 cell lines (54%) displayed IC_90_s > 5 μM. There were clear differences between the individual cancer entities. In leukaemia/lymphoma 40 (82%) out of 49 cell lines, in multiple myeloma 10 (100%) out of 10 cell lines, and in neuroblastoma 28 (88%) out of 32 cell lines displayed an IC_90_ < 1 μM. Together, these three entities accounted for 78 (67%) of the 117 cell lines that displayed IC_90_s < 1 μM among the 26 cancer entities. In all other entities, except Ewing's sarcoma (4 (57%) out of 7 cell lines with an IC_90_ < 1 μM) and head and neck cancer (3 (60%) out of 5 cell lines with an IC_90_ < 1 μM), the majority of the cell lines displayed IC_90_s > 1 μM. None of the 9 gastric cancer, the 13 melanoma, the 6 oesophageal cancer, the 10 ovarian cancer, the 10 pancreas cancer, the 5 prostate cancer, or the 3 retinoblastoma cell lines displayed an IC_90_ < 1 μM (Fig. 1; Suppl. Table S1).

### Effects of flubendazole on cancer cell viability in a panel of 140 drug-resistant neuroblastoma cell lines

**Figure 2:**
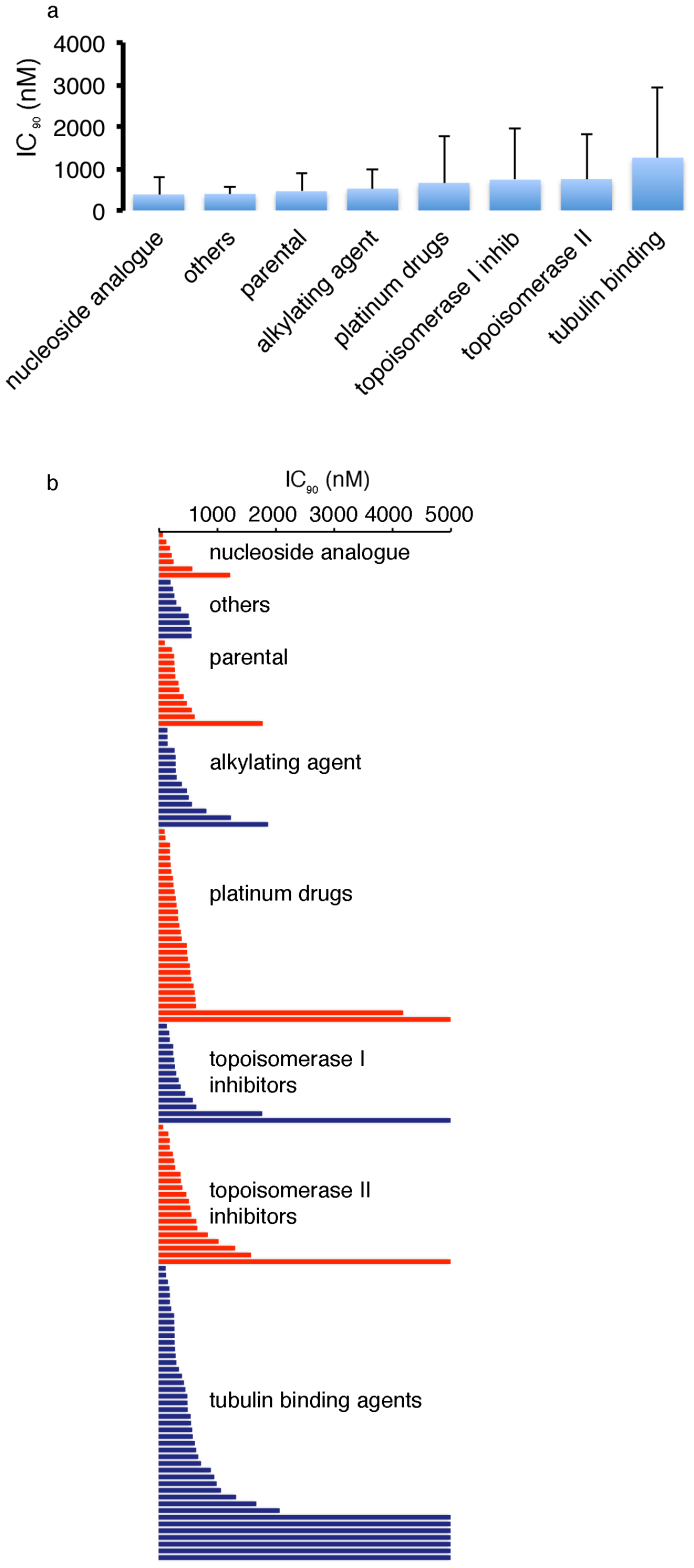
Effects of flubendazole in a panel of parental neuroblastoma cell lines and their sub-lines adapted to anti-cancer drugs. The flubendazole concentrations that decreased cancer cell viability by 90% (IC_90_) were determined by MTT assay after 5 days of incubation (flubendazole 5 μM was the maximum concentration tested). (a) The average IC_90_ values for each cancer entity are presented. Error bars represent a single standard deviation. (b) The average IC_90_ for each cell line (average from 2 experiments per cell line) is presented. (c) The Wilcoxon rank sum test did not indicate a significant difference (p < 0.05) between IC_90_s for the cell lines with acquired resistance to different drug classes. The detailed data is presented in Suppl Table S2.

Acquired resistance formation is a major problem in high-risk neuroblastoma^11^. 119 (85%) out of 140 drug-resistant neuroblastoma cell lines displayed flubendazole IC_90_s < 1 μM, 11 cell lines (8%) displayed IC_90_s > 1 μM and <5 μM, and 10 cell lines (7%) displayed IC_90_s > 5 μM (Fig. 2, Suppl. Fig. S2, Suppl. Table S2).

The majority (87, 62%) of the 140 drug-resistant neuroblastoma cell lines was similarly sensitive to flubendazole like the corresponding parental cell lines as indicated by fold changes IC_90_ drug-resistant sub-line/IC_90_ respective parental cell line between 0.5 and 2.0. In 39 (28%) of the drug-resistant cell lines the fold change was >2.0. In 14 drug-resistant cell lines it was <0.5 (Fig. 2, Suppl. Table S2).

In concordance with its anticipated effects on microtubule function^2^, flubendazole induced a G2/M arrest in neuroblastoma cells (Suppl. Fig. S3). The tubulin-binding agents can be divided into destabilising agents that inhibit tubulin polymerisation and stabilising agents that inhibit microtubule degradation in super-therapeutic concentrations^12^,^13^. The taxanes docetaxel, paclitaxel, and cabazitaxel are stabilising agents that target the taxoid domain. The *Vinca* alkaloids vinblastine, vincristine, and vinorelbine are destabilising agents that target the vinca domain. Flubendazole and 2-methoxyestradiol are destabilising agents that target the colchicine domain^2^,^12^,^13^. The neuroblastoma cell lines with acquired resistance to tubulin-binding agents displayed the highest average IC_90_ and IC_50_ values for flubendazole (Fig. 2, Suppl. Fig. 2, Suppl. Table S2) but the differences did not reach statistical significance. 12 (80%) of the 15 taxane-resistant cell lines showed a similar flubendazole sensitivity like the corresponding parental cells (IC_90_ resistant cell line/IC_90_ corresponding parental cell line >0.5 and <2.0). 2 (13%) taxane resistant cell lines showed an increased flubendazole resistance (IC_90_ resistant cell line/IC_90_ corresponding parental cell line >2.0) and 1 cell line showed enhanced sensitivity (IC_90_ resistant cell line/IC_90_ corresponding parental cell line <0.5). 12 (43%) of the 28 *Vinca* alkaloid-resistant cell lines displayed cross-resistance to flubendazole while the remaining 16 (57%) *Vinca* alkaloid-resistant cell lines were similarly sensitive to flubendazole like the corresponding parental cells. The 2-methoxyestradiol-resistant cell line UKF-NB-3^r^ME^1000^ displayed an 8-fold increased flubendazol IC_90_ relative to UKF-NB-3. These data suggest that cross-resistance formation to flubendazole is more likely in cell lines resistant to destabilising agents than to stabilising agents (Fig. 2, Suppl. Fig. S2, Suppl. Table S2).

### Effects of flubendazole on primary neuroblastoma cells

**Figure 3:**
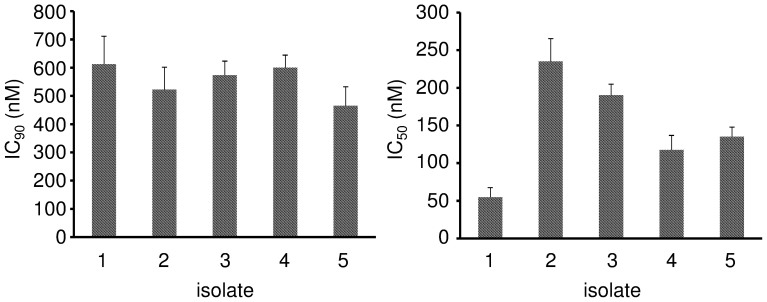
Effects of flubendazole on primary neuroblastoma cells. The flubendazole concentrations that decreased cancer cell viability by 90% (IC_90_) and 50% (IC_50_) were determined by MTT assay after 5 days of incubation. Values are expressed as mean ± standard deviation.

Primary neuroblastoma cells isolated from the bone marrow of five patients with metastasised INSS stage 4 disease showed similar flubendazole sensitivity like the investigated neuroblastoma cell lines. The IC_90_ values ranged from 464.7 to 612.2 nM (Fig. 3, Suppl Table S3).

### Effects of ABCB1 or ABCG2 expression on cancer cell sensitivity to flubendazole

The anti-cancer effects of many anti-cancer drugs are affected by the expression of ATP-binding cassette (ABC) transporters including ABCB1 and ABCG2^14^,^15^. UKF-NB-3 cells were transduced with lentiviral vectors encoding for ABCB1 or ABCG2, respectively, as described previously^16^,^17^. ABCB1 expression enhanced UKF-NB-3 cell resistance to the ABCB1 substrate vincristine but not to flubendazole (Suppl. Table S4). The ABCB1 inhibitor verapamil sensitised ABCB1-expressing neuroblastoma cells to vincristine but not to flubendazole. Similarly, ABCG2 expression increased UKF-NB-3 cell resistance to the ABCG2 substrate mitoxantrone but not to flubendazole. The ABCG2 inhibitor WK-X-34^18^ sensitised ABCG2-expressing UKF-NB-3 cells to mitoxantrone but not to flubendazole (Suppl. Table S4).

### Role of p53 signalling in the flubendazole-mediated anti-cancer effects

**Figure 4:**
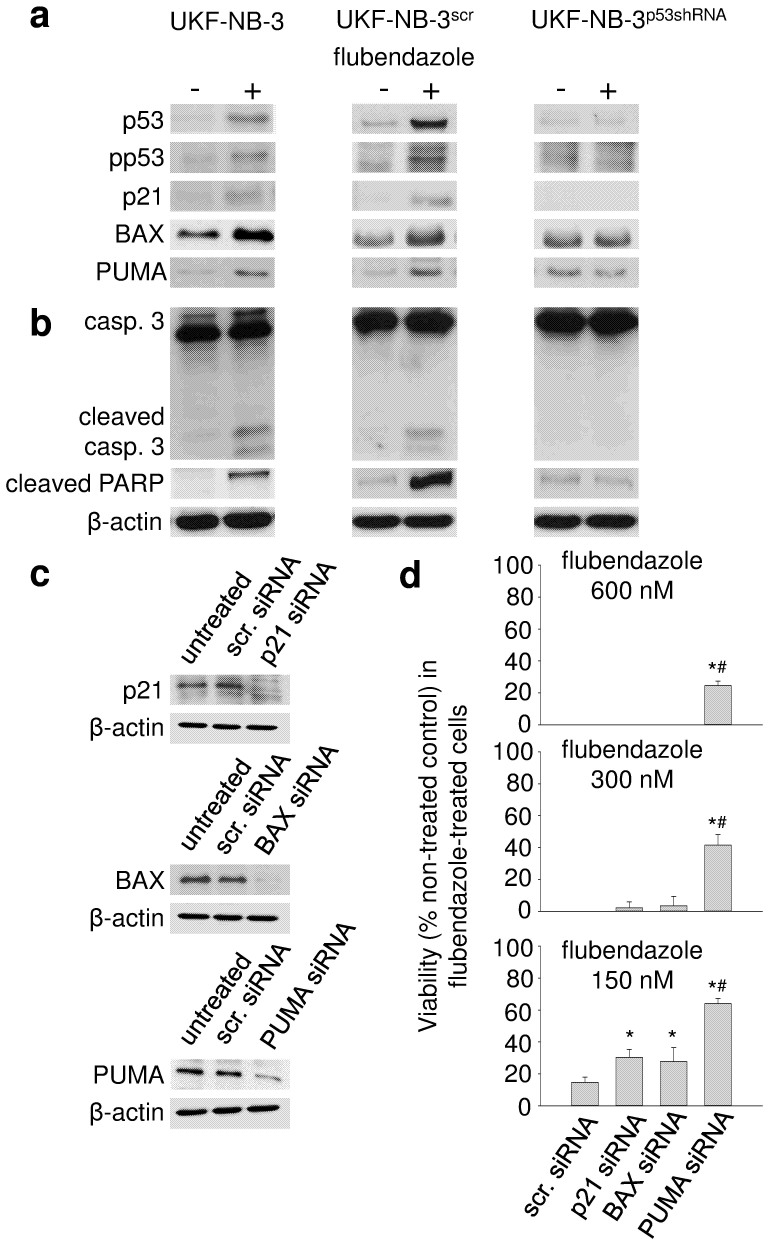
Effects of flubendazole on p53 activation and apoptosis in UKF-NB-3 cells. UKF-NB-3 cells, UKF-NB-3 cells transduced with a lentiviral vector encoding for p53 shRNA (UKF-NB-3^p53shRNA^), and UKF-NB-3 cells transduced with a control vector (UKF-NB-3^scr^) were incubated for 24 h with flubendazole 300 nM. (a) Western blots indicating the expression of p53 and its target gene products p21, BAX, and PUMA; (b) Western blots indicating caspase 3 (casp. 3) cleavage/activation and cleaved PARP; (c) Western blots indicating siRNA-mediated depletion of p21, BAX, or PUMA in UKF-NB-3 cells (scr. siRNA, non-targeted scrambled siRNA) 24 h post transfection; (d) Influence of the siRNA-mediated depletion of p21, BAX, or PUMA on the viability of UKF-NB-3 cells in the presence of flubendazole. 48 h post transfection with siRNA, cells were transferred into 96-well plates in the absence or presence of serial dilutions of flubendazole. The cell viability was determined after a 5 day incubation period by MTT assay. * P < 0.05 relative to scr. siRNA; ^#^ P < 0.05 relative to p21 and BAX siRNA Original Western blots corresponding to Figure 4A, 4B, and 4C are displayed in Suppl. Fig. S7.

Cell death induced by many anti-cancer drugs involves p53 activation^19^. Flubendazole induced the expression of the p53 target genes *CDKN1A* (encodes for p21), *BAX*, and *BBC3* (encoding for PUMA) (Fig. 4a) and signs of apoptosis (caspase 3 activation, PARP cleavage) (Fig. 4b) in UKF-NB-3 cells. Flubendazole-treated p53-depleted UKF-NB-3 (UKF-NB-3^p53shRNA^) cells showed a lack of flubendazole-induced p53 target gene expression (Fig. 4a), and reduced flubendazole-induced caspase 3 activation and PARP cleavage (Fig. 4b). Also, flubendazole induced a stronger increase of the sub-G1 fraction in wild-type p53-expressing than in p53-depleted cells (Suppl. Fig. S4). Different p53 downstream molecules appear to be involved in the flubendazole-induced effects. SiRNA-mediated depletion of Bax, p21, and PUMA impaired the flubendazole sensitivity of UKF-NB-3 cells with PUMA depletion causing the strongest effects (Fig. 4c and d). Moreover, siRNA-mediated depletion of Bax, p21, and PUMA inhibited flubendazole-induced caspase 3/7 activation (Suppl. Fig. S5). Again, PUMA depletion resulted in the most pronounced effects. Caspase 9 depletion that served as control also reduced caspase 3/7 activation (Suppl. Fig. S5).

**Figure 5:**
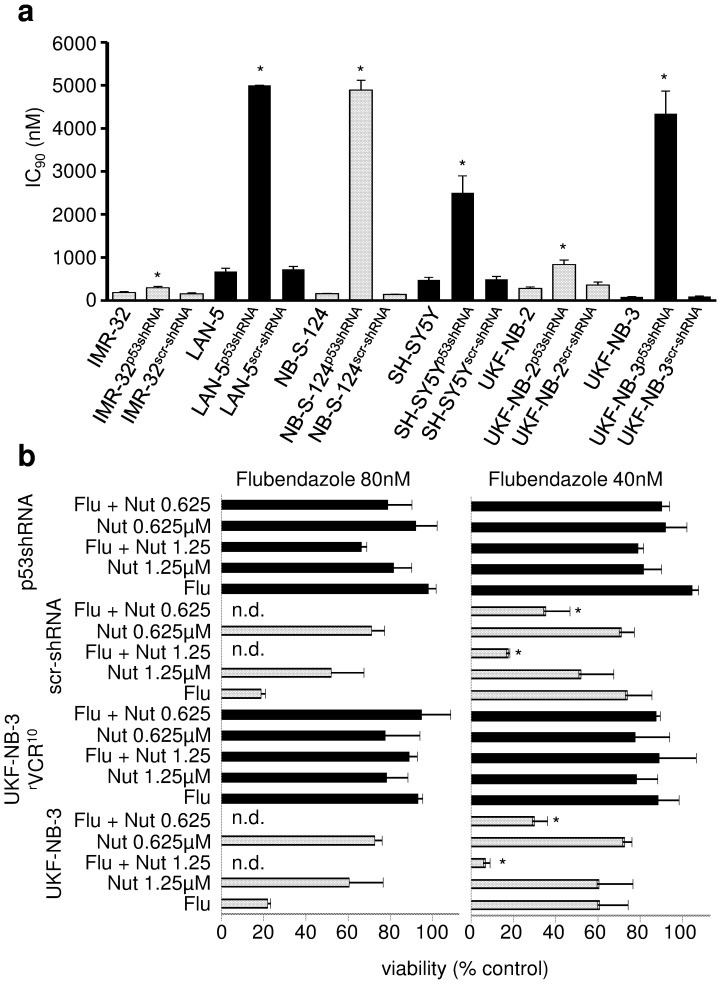
Role of p53 signalling in the anti-neuroblastoma effects of flubendazole. (a) Flubendazole IC_90_ values in control cell lines and neuroblastoma cells transduced with lentiviral vectors encoding for shRNA directed against p53 (p53shRNA) or scrambled, non-targeting shRNA (scr-shRNA). * P < 0.05 relative to non-transduced cell line; (b) Viability of p53 wild-type UKF-NB-3 cells, p53-mutated UKF-NB-3^r^VCR^10^cells, UKF-NB-3 cells transduced with a lentiviral vector encoding scrambled, non-targeting shRNA (scr-shRNA), or UKF-NB-3 cells transduced with a lentiviral vector encoding shRNA directed against p53 (p53shRNA) after treatment with flubendazole (Flu), nutlin-3 (Nut, μM), or their combination as indicated by MTT assay after 5 day incubation. * P < 0.05 relative to either single treatment, n.d. = not detectable.

Moreover, p53 depletion in IMR-32, UKF-NB-2, UKF-NB-3, LAN-5, NB-S-124, and SH-SY5Y cells resulted in decreased flubendazole sensitivity (Fig. 5a, Suppl. Table S5).

The MDM2 inhibitor nutlin-3^20^ enhanced the effects of flubendazole in p53 wild-type UKF-NB-3 cells (Fig. 5b; Suppl. Table S6) but did not affect the effects of flubendazole in p53-mutated UKF-NB-3^r^VCR^10^ cells and hardly influenced the effect of flubendazole in p53-depleted UKF-NB-3^p53shRNA^ cells (Suppl. Table S6). Some combined effects may be observed in UKF-NB-3^p53shRNA^ cells treated with the combination of flubendazole 80 nM and nutlin-3 1.25 μM. This is most likely caused by some remaining p53 activity due to incomplete p53 depletion.

### In vivo-evaluation of flubendazole in the chorioallantoic membrane (CAM) assay

To develop DMSO-free flubendazole preparations we developed flubendazole-cyclodextrin complexes. (2-hydroxypropyl)-β-CD increased the aqueous solubility of flubendazole while γ-CD did not. A 20% (m/V) (2-hydroxypropyl)-β-CD formulation containing 74 μM flubendazole and 0.1% saline was similar active against neuroblastoma cells like flubendazole in DMSO and then diluted with cell culture medium (Suppl. Table S7). The preparation was stable during storage for eight weeks as indicated by HPLC and assessment of biological activity (Suppl. Table S7).

**Figure 6:**
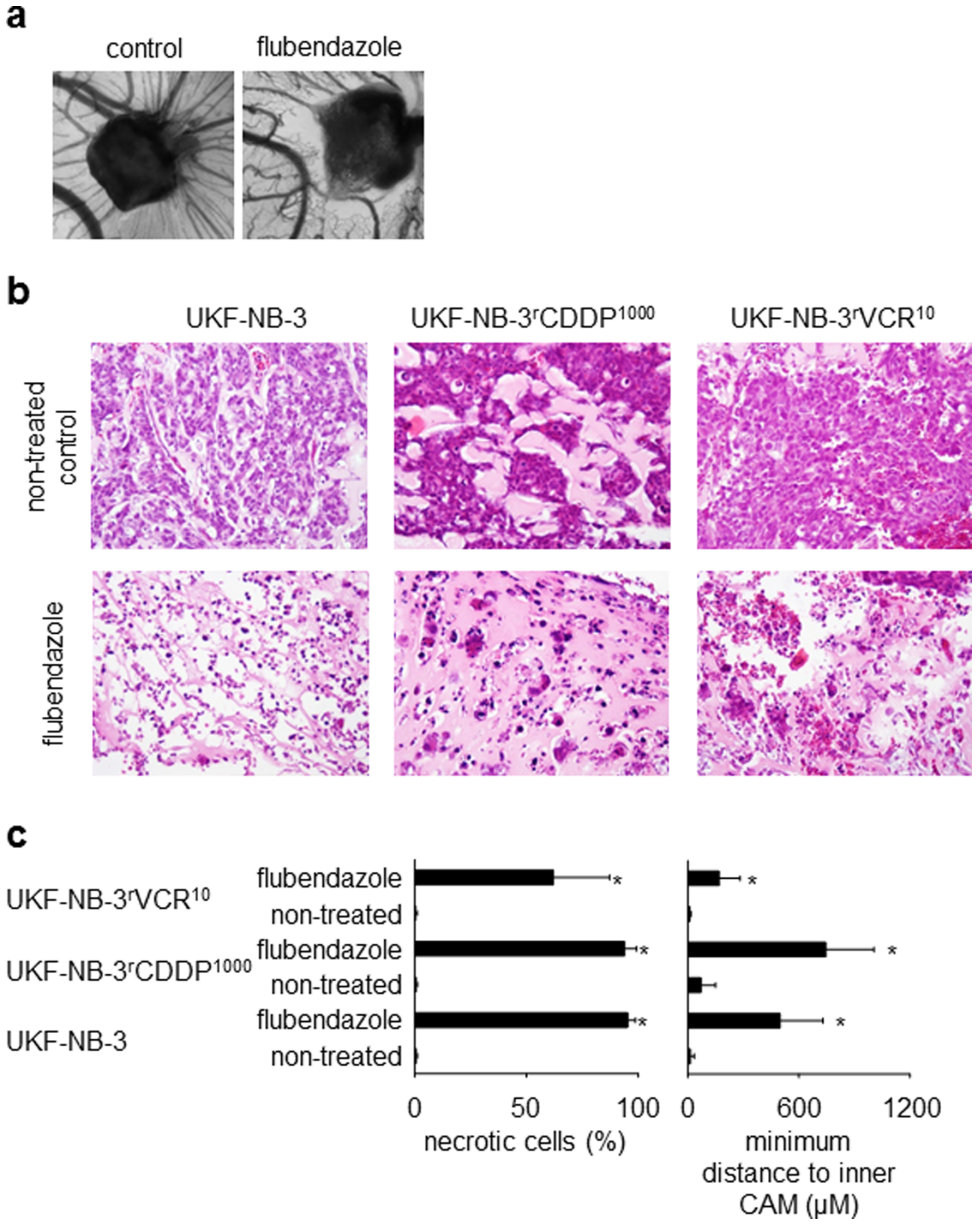
In vivo-effects of flubendazole in the chick chorioallantoic membrane (CAM) model. Flubendazole was applied as water-soluble (2-hydroxypropyl)-β-cyclodextrin complex. (a) Representative photographs demonstrating the effects of flubendazole (20 μg/mL) on vessel formation in the CAM compared to a vehicle-treated control; (b) haematoxylin/eosin staining of representative CAMs demonstrating the effects of flubendazole (2 μg, applied as (2-hydroxypropyl)-β-cyclodextrin complex in 100 μL 0.9% NaCl solution) (original magnification: 20-fold). Flubendazole-treated tumours grow in a less extensive manner and present with large necrotic areas. (c) Quantification of necrosis formation and cancer cell invasion in the CAM was studied in UKF-NB-3 (5 non-treated tumours, 8 flubendazole-treated tumours), UKF-NB-3^r^CDDP^1000^ (4 non-treated tumours, 8 flubendazole-treated tumours), and UKF-NB-3^r^VCR^10^ (9 non-treated tumours, 9 flubendazole-treated tumours). To measure cancer cell invasion, the minimum distance of the cancer cell invasion front to the inner CAM border was determined. Higher values indicate a greater distance to the inner membrane and, therefore, lower invasion. * P < 0.05 relative to non-treated control.

For the investigation of vessel formation, the CAM assay was performed using drug-loaded gelatin sponges as described previously^21^. Vessel formation was scored from 0 (complete suppression of vessel formation) to 5 (vessel formation using non-treated control sponges). The analysis of vessel formation surrounding 10 vehicle-treated control sponges resulted in a vessel formation score of 4.5 ± 0.7. The analysis of vessel formation surrounding 10 flubendazole-treated sponges resulted in a vessel formation score of 0.7 ± 0.8 (P = 1 × 10^−6^ relative to vehicle-treated control sponges). Representative photographs are presented in Fig. 6a.

To analyse the effects of flubendazole on neuroblastoma xenograft growth in the CAM, three cell lines were used. UKF-NB-3 (flubendazole IC_90_ = 102.3 ± 12.7 nM) and its cisplatin-resistant sub-line UKF-NB-3^r^CDDP^1000^ (flubendazole IC_90_ = 122.1 ± 16.7 nM) were selected as highly flubendazole-sensitive cell lines. The vincristine-resistant UKF-NB-3 sub-line UKF-NB-3^r^VCR^10^ (flubendazole IC_90_ = 552.3 ± 60.9 nM) had displayed an approximately five-fold decreased flubendazole sensitivity compared to UKF-NB-3 and UKF-NB-3^r^CDDP^1000^. Flubendazole 2 μg (applied as 100 μL of a 20 μg/mL solution) treatment resulted in all cell lines in less extensive growth and large necrotic areas. Representative photographs are presented in Fig. 6b and Suppl. Fig. S6.

To quantify the drug effects, the fractions of necrotic cells were determined and cancer cell invasion was analysed. The flubendazole-induced necrosis levels were higher in UKF-NB-3 and UKF-NB-3^r^CDDP^1000^ tumours than in UKF-NB-3^r^VCR^10^ tumours although differences did not reach statistical significance (Fig. 6c). To measure cancer cell invasion, the minimum distance of the cancer cell invasion front to the inner CAM border was determined. Higher values indicate a greater distance to the inner membrane and, therefore, lower penetration. Again, flubendazole exerted stronger effects on UKF-NB-3 and UKF-NB-3^r^CDDP^1000^ tumours than on UKF-NB-3^r^VCR^10^ tumours (Fig. 6c).

## Discussion

Flubendazole had been demonstrated to exert activity against leukaemia and myeloma cells^2^. Here, a screen of flubendazole in 321 cancer cell lines from 26 entities confirmed the flubendazole-sensitivity of leukaemia and multiple myeloma cells and identified neuroblastoma as potential additional flubendazole-sensitive cancer entity. 140 neuroblastoma cell lines and primary neuroblastoma samples from five different patients were similarly sensitive to flubendazole. 119 (85%) out of 140 neuroblastoma cell lines and all five primary neuroblastoma isolates displayed IC_90_ values <1000 nM. Only in two further cancer entities (Ewing's sarcoma, head and neck cancer), a substantial fraction of the investigated cell lines was sensitive to flubendazole in concentrations <1 μM that appear to be pharmacologically achievable^8^. Notably, a number of the entities that were in general regarded to be insensitive to flubendazole included flubendazole-sensitive cell lines emphasising the need for markers indicating cancer cell sensitivity to flubendazole.

Neuroblastoma is the most frequent extracranial solid childhood tumour. About half of patients suffer from high-risk disease associated with overall survival rates below 50% despite intensive therapy^22^. Resistance acquisition is a major problem in neuroblastoma^11^. Among 140 neuroblastoma cell lines with acquired resistance to a range of anti-cancer drugs, 119 cell lines (85%) displayed IC_90_ values below 1 μM. 87 drug-resistant neuroblastoma cell lines (62%) were similar sensitive to flubendazole like the corresponding parental cells. 39 drug-resistant cell lines (28%) showed cross-resistance to flubendazole, 14 drug-resistant cell lines (10%) were more sensitive to flubendazole than their parental counterparts.

Flubendazole has been described to interfere with microtubule function^2^. There are different classes of tubulin-binding agents. In super-therapeutic concentrations, destabilising agents inhibit tubulin polymerisation, stabilising agents microtubule degradation^12^,^13^. Our panel of resistant neuroblastoma cell lines contained cell lines resistant to the taxanes docetaxel, paclitaxel, and cabazitaxel (stabilising agents, target the taxoid domain), to the *Vinca* alkaloids vinblastine, vincristine, and vinorelbine (destabilising agents, target the vinca domain), and to 2-methoxyestradiol that is like flubendazole a destabilising agents that targets the colchicine domain^2^,^12^,^13^. The neuroblastoma cell lines with acquired resistance to tubulin-binding agents displayed the highest average flubendazole IC_90_ and IC_50_ values but the differences did not reach statistical significance. These findings suggest that acquired resistance to a tubulin-binding agent may not necessarily be associated with a substantially decreased sensitivity to flubendazole. In concordance, albendazole, another benzimidazole anthelminthic, had been shown to be active in cancer cell lines resistant to the stabilising tubulin-binding agents paclitaxel and epothilone B^23^,^24^. Also, albendazole and flubendazole had been shown to increase the anti-cancer effects of paclitaxel and vinblastine^2^,^25^.

In concordance with previous results^2^, the anti-cancer effects of flubendazole were not impaired by ABCB1 expression. Moreover, ABCG2, another major ABC transporter^14^,^15^, did not affect flubendazole efficacy. However, flubendazole appears to exert its anti-neuroblastoma effects in part via p53 activation. Flubendazole induced p53 signalling, displayed a strongly enhanced potency in combination with the MDM2 inhibitor and p53 activator nutlin-3, and the flubendazole activity was substantially reduced in the absence of functional p53. RNAi-mediated depletion experiments suggested PUMA to be a critical mediator of the flubendazole-induced anti-neuroblastoma effects. Since inactivation of p53 signalling has been suggested as an acquired resistance mechanism in neuroblastoma^11^, this may be of clinical relevance. While p53 was found mutated in only 2% of neuroblastomas at diagnosis, p53 mutations were detected in about 15% of neuroblastomas at relapse^11^,^26^,^27^. In concordance, different cell line-based studies pointed towards a role of p53 inactivation as (acquired) resistance mechanism in neuroblastoma^11^,^28^,^29^,^30^,^31^,^32^,^33^. Therefore, flubendazole appears to be a promising treatment option in particular for the majority of p53 wild-type neuroblastomas.

The poorly water-soluble flubendazole was administered in 0.9% NaCl and 0.01% Tween-80 in a previous study^2^. We were not successful in preparing a suitable flubendazole preparation using this method. Acidic β-CD complexes of flubendazole for oral use that had been described before^8^,^34^ not be suited for parenteral application or the CAM assay. Therefore, we developed a (2-hydroxypropyl)-β-CD preparation of flubendazole that was equally effective as DMSO-dissolved flubendazole in cell culture. This flubendazole (2-hydroxypropyl)-β-CD preparation inhibited vessel and tumour formation in the chick chorioallantoic membrane. These findings are in concert with reports that demonstrated anti-leukaemia and anti-myeloma activity of flubendazole in vivo^2^. In addition, we provide evidence that flubendazole also exerts anti-angiogenic effects.

Mebendazole and albendazole are further benzimidazole anthelminthic agents that have been shown to exert anti-cancer effects^1^,^3^,^4^,^5^,^6^,^7^,^35^,^36^,^37^,^38^,^39^. Recent results suggested that mebendazole and albendazole differ significantly in their anti-cancer mechanisms of action^7^. Based on a comparison of flubendazole and mebendazole in a panel of 39 cancer cell lines from 13 cancer entities (Suppl. Table 8) and in 25 drug-resistant neuroblastoma cell lines (Suppl. Table 9) these two compounds appear to exert similar anti-cancer effects.

In conclusion, we show that the well-tolerated anthelminthic flubendazole^40^,^41^,^42^, represents a potential treatment option for neuroblastoma, in particular for the majority of neuroblastomas with functional p53.

## Methods

### Drugs

WK-X-34 was synthesised as described before^18^. Verapamil and flubendazole were purchased from Sigma Aldrich (Taufkirchen, Germany). Nutlin-3 was purchased from Selleck Chemicals via BIOZOL GmbH (Eching, Germany).

### Cells

The origins of the investigated parental cell lines and the cell culture media are shown in Suppl. Table S1.

Parental chemosensitive cell lines were adapted to growth in the presence of anti-cancer drugs by continuous exposure of these cells to increasing drug concentrations as described previously^28^,^29^,^43^. The drug-resistant cell lines were derived from the resistant cancer cell line (RCCL) collection (www.kent.ac.uk/stms/cmp/RCCL/RCCLabout.html) (Suppl. Table S10).

Cells were routinely tested for mycoplasma contamination and authenticated by short tandem repeat or variable number tandem repeat profiling.

p53-depleted cells or cells showing high expression of ABCB1 (also known as MDR1, gene product also known as P-glycopotein) or ABCG2 (also known as BCRP) were established as described previously^17^ using the Lentiviral Gene Ontology (LeGO) vector technology^44^,^45^; www.lentigo-vectors.de).

Fresh neuroblastoma cells (MYCN amplified) were isolated from the bone marrow aspirate of five patients with metastasised INSS stage 4 neuroblastoma following informed consent. Primary cells were cultivated in IMDM supplemented with 10% FCS, 100 IU/ml penicillin, and 100 mg/ml streptomycin at 37°C.

### Viability assay

Cell viability was tested by the 3-(4,5-dimethylthiazol-2-yl)-2,5-diphenyltetrazolium bromide (MTT) dye reduction assay after 120 h incubation modified as described previously^16^,^29^.

Cell viability was tested in 96-well plates using 1 in 4 dilution steps. The maximum flubendazole concentration tested was 5000 nM. IC_50_ and IC_90_ values were determined using the CalcuSyn software (Biosoft, Cambridge, UK). Differences in the IC_50_ and IC_90_ values between the cancer entities were tested using the Wilcoxon rank sum test^9^ with subsequent correction for multiple testing by the Benjamini-Hochberg method^10^.

### Western blot

Cells were lysed in Triton X-sample buffer and separated by SDS-PAGE. Proteins were detected using specific antibodies directed against β-actin (BioVision via BioCat GmbH, Heidelberg, Germany), p21, Bax, cleaved PARP, PUMA, caspase 3, caspase 9 (all from Cell Signaling via New England Biolabs, Frankfurt am Main, Germany), p53 (Enzo Life Sciences, Lörrach, Germany), and phosphorylated p53 (serine 15) (Millipore, Schwalbach, Germany) and were visualised by enhanced chemiluminescence using a commercially available kit (Amersham, Freiburg, Germany).

### RNA interference experiments

Synthetic siRNA oligonucletides targeting BAX, PUMA, p21, or Caspase 9 and control non target siRNAs (ON-TARGETplus SMARTpool) were purchased from Dharmacon (Lafayette, CO, USA). The target sequences are presented in Suppl. Table S11. Transfections were performed using the Neon^TM^ Transfection System (Invitrogen, Darmstadt, Germany) according to the manufacturer's protocol. 2 × 10^6^ UKF-NB-3 cells were suspended in 200 μl re-suspension buffer containing 2.5 μM siRNA. After electroporation (voltage 1400, width 20, pulses 2), the cells were transferred into fibronectin (100 μg/ml)-coated 6-well-plates containing pre-warmed IMDM plus 10 % FCS. Western blot analyses were performed and cells were used for viability assays 48 h post transfection.

### Caspase 3/7 activity assay

The activity of the caspases 3 and 7 was examined using the Caspase-Glo® 3/7 Assay (Promega GmbH, Mannheim, Germany) following the manufacturer's instructions. Cells were seeded in 96-well cell culture plates and allowed to adhere overnight. After drug treatment, the culture plates were adjusted to room temperature. Then, the cells were incubated for 5 min with the pre-mixed substrate and the luminescent signal was measured with a plate reader (Tecan, Crailsheim, Germany) for 30 cycles.

### Development of water-soluble flubendazole preparations

5.0 mg flubendazole were suspended in 10.0 mL aqueous solutions of the cyclodextrin (CD) derivatives (2-hydroxypropyl)-β-CD (0.2–30% m/V, Cargill, Krefeld, Germany) or γ-CD (1–20% m/V, Wacker-Chemie, Munich, Germany). After vortexing and ultrasonication (15 min), the samples were stirred at room temperature for 5 days. After 48 h and 120 h samples were taken, filtered through pre-saturated cellulose acetate membrane filters (0.45 μm), diluted with purified water and analysed by HPLC for the flubendazole concentration using an isocratic mixture of water and methanol (31:69) containing 0.1% trifluoracetic acid at a temperature of 30°C and a reverse phase column (Kinetex PFP, 2.6 μm, 100 × 4.6 mm) in combination with a KrudKatcher™ Ultra In-line filter (Phenomenex, Aschaffenburg, Germany) in an Agilent 1200 HPLC system (Agilent, Böblingen, Germany) with DAD detection (flow rate 1.0 mL/min). Flubendazole was detected at 234 nm (retention time 2.1 min).

Validation of the HPLC analysis was performed according to the ICH harmonised tripartite guideline “Validation of analytical procedures: Text and Methodology Q2(R1)” (www.ich.org). An acceptable degree of linearity, accuracy and precision was confirmed for concentrations between 0.25 and 1 μg/mL. The limit of detection was <0.01 μg/mL; the limit of quantification was >0.2 μg/mL.

### Chorioallantoic membrane (CAM) assay

The effects of flubendazole on vessel formation and in vivo tumour growth were investigated in the chorioallantoic membrane (CAM) assay. For the investigation of vessel formation the CAM assay was performed using drug-loaded gelatin sponges as described previously^21^. Sponges were placed onto the CAM at day 8. Vessel formation was determined at day 12. 20% Luconyl Black in phosphate-buffered saline was injected into a vitelline vein. Vessel formation was scored from 0 (complete suppression of vessel formation) to 5 (vessel formation using non-treated control sponges).

Tumour growth in the CAM was examined following described methods^46^. Briefly, 5 × 10^6^ cells were suspended in 30 μl extracellular matrix (Growth-factor reduced Matrigel, BD Biosciences, Heidelberg, Germany) and implanted on the CAM of fertilised chicken eggs on day 11 of embryo development. Eggs were incubated for another 3 days to allow formation of a distinct tumour mass. On day 14, a small silicone ring was placed around the tumour mass and drugs were administered. On day 18, the tumours were sampled with the surrounding CAM, fixed using 4% paraformaldehyde, and embedded in paraffin. Sections (4 μM) were haematoxylin/eosin-stained.

### Statistics

Results are expressed as mean ± S.D. of at least three experiments. Comparisons between two groups were performed using Student's t-test. Three and more groups were compared by ANOVA followed by the Student-Newman-Keuls test. P values lower than 0.05 were considered to be significant.

## Author Contributions

Ma.M. and J.C. jr. designed the study, performed experiments, analysed data, and wrote the manuscript. B.A., F.R., N.L., Y.V., Mi.M., T.S., P.N.H., K.R., B.A.A., S.F., S.S., K.L., W.G.D., R.Z. and J.C. performed experiments, analysed data, and proofread the manuscript. M.W. and F.W. provided essential materials, analysed data, and proofread the manuscript. M.N.W. performed statistical analyses, analysed data, wrote parts of the manuscript, and proofread the manuscript.

## Supplementary Material

Supplementary Information Suppl Files
