# Supplementary material for: Identification of flubendazole as potential anti-neuroblastoma compound in a large cell line screen
Source: Sci Rep. 2015 Feb 3;5:8202. doi: 10.1038/srep08202 (PMC4314641; doi:10.1038/srep08202)
Supplement: Supplementary Information — Suppl Files [file srep08202-s1.pdf]

Supplementary files to:

Identification of flubendazole as potential anti-neuroblastoma compound in a large cell line screen

Martin Michaelis, Bishr Agha, Florian Rothweiler, Nadine Löschmann, Yvonne Voges, Michel Mittelbronn, Tatjana Starzetz, Patrick N. Harter, Behnaz A. Abhari, Simone Fulda, Frank Westermann, Kristoffer Riecken, Silvia Spek, Klaus Langer, Michael Wiese, Wilhelm G. Dirks, Richard Zehner, Jaroslav Cinatl, Mark Wass, Jindrich Cinatl jr.

Suppl. Figure S1

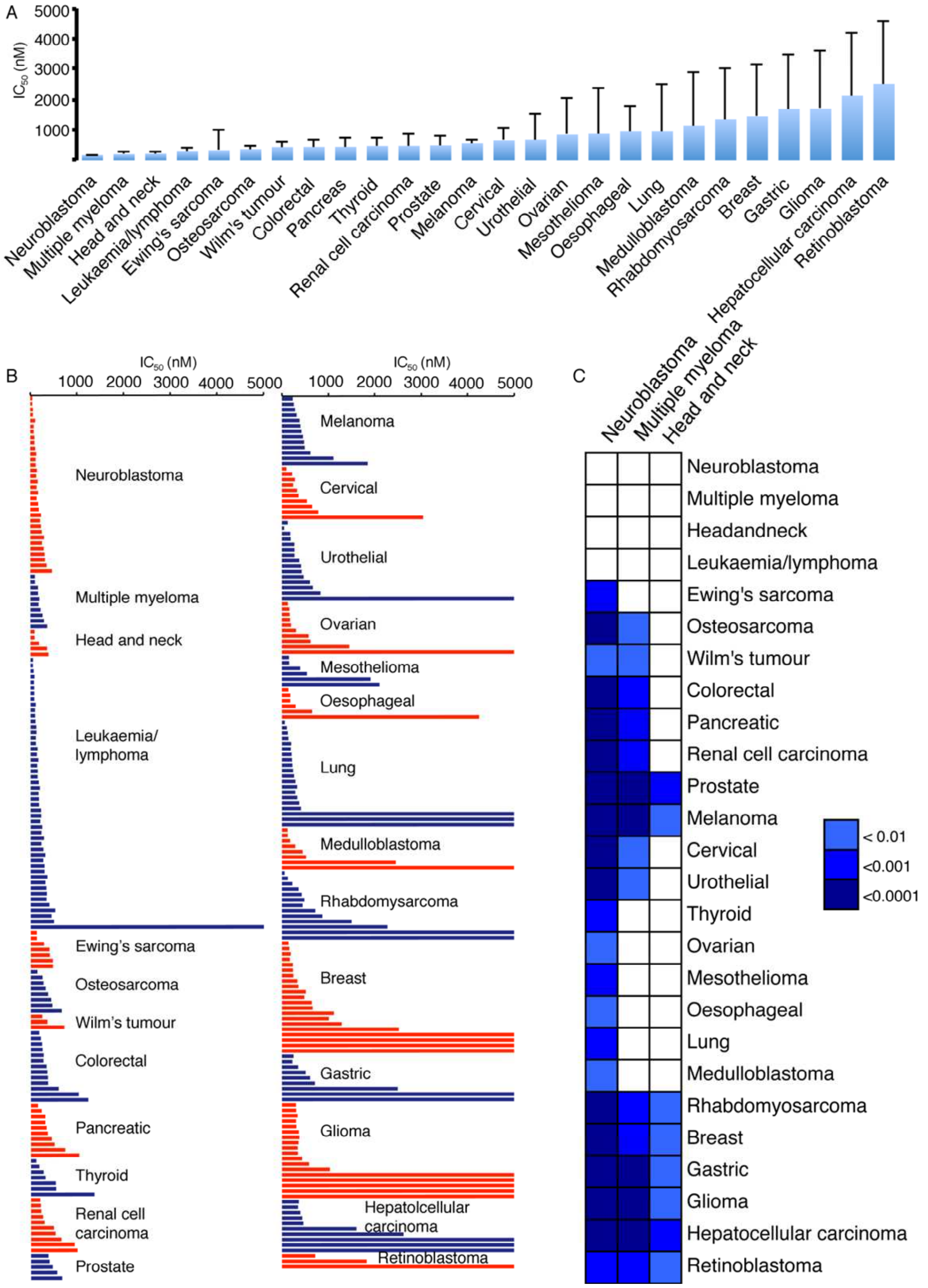

Suppl. Figure S1. Effects of flubendazole in a panel of cancer cell lines from different entities. The flubendazole concentrations that decreased cancer cell viability by 50% (IC_{50}) were determined by MTT assay after 5 days of incubation (flubendazole 5µM was the maximum concentration tested). A) The average IC_{90} values for each cancer entity are presented. Error bars represent a single standard deviation B) The average IC_{50} for each cell line (average from 2 experiments per cell line) is presented. Cancer entities are ordered by increasing overall average as shown in A. C) The Wilcoxon rank sum test indicated significant differences ($p < 0.05$) between IC_{50}s for the different cancer entities. Cells are coloured according to their significance level after multiple testing correction applying the Benjamini-Hochberg method. The detailed data is shown in Suppl. Table S1.

Suppl. Figure S2

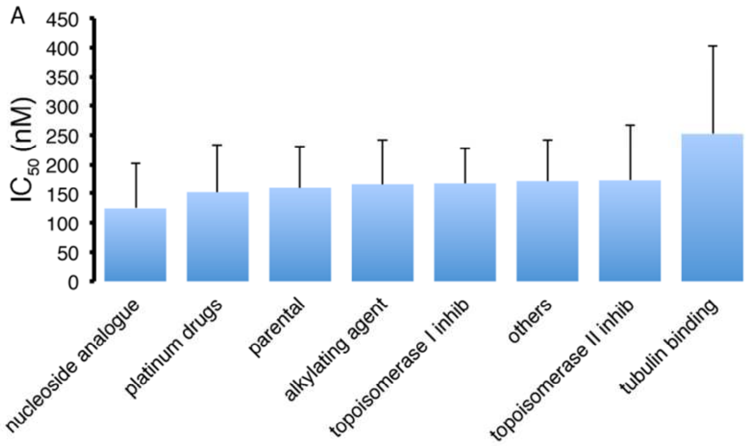

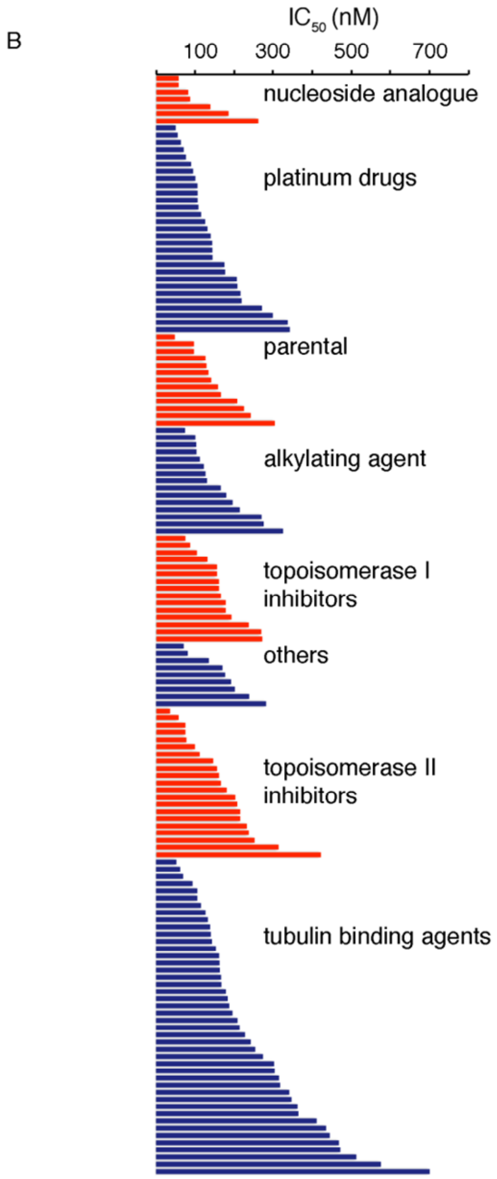

Suppl. Figure S2. Effects of flubendazole in a panel of parental neuroblastoma cell lines and their sub-lines adapted to anti-cancer drugs. The flubendazole concentrations that decreased cancer cell viability by 50% (IC_{50}) were determined by MTT assay after 5 days of incubation (flubendazole 5µM was the maximum concentration tested). A) The average IC_{50} values for each cancer entity are presented. Error bars represent a single standard deviation. B) The average IC_{50} for each cell line (average from 2 experiments per cell line) is presented. The Wilcoxon rank sum test to test did not indicate a significant difference ($p < 0.05$) between IC_{50}s for the cell lines with acquired resistance to different drug classes. The detailed data is presented in Suppl Table S2.

Suppl. Figure S3

UKF-NB-3^{scr}

Control

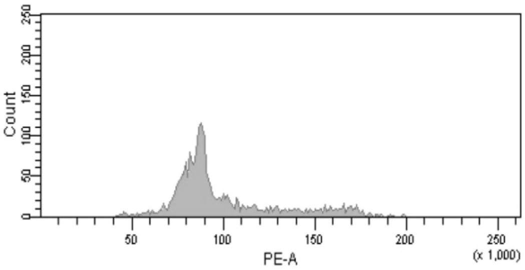

Flubendazole 0.2µM

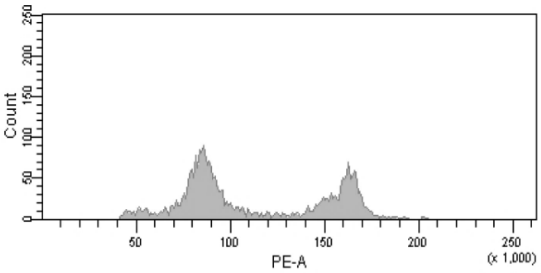

UKF-NB-3^{p53shRNA}

Control

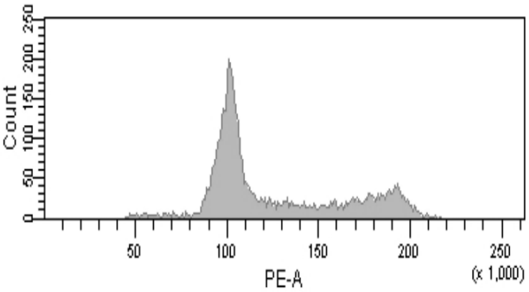

Flubendazole 0.8µM

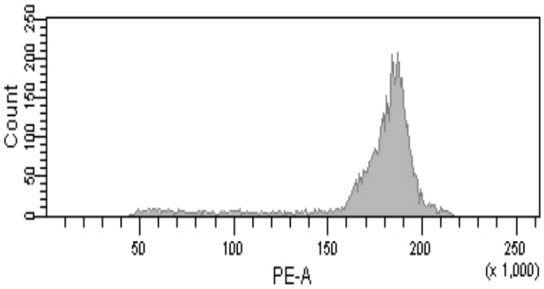

NB-S-124^{scr}

Control

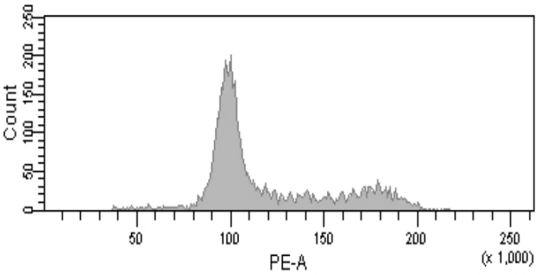

Flubendazole 0.15µM

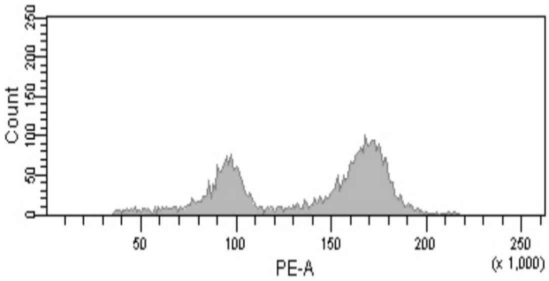

NB-S-124^{p53shRNA}

Control

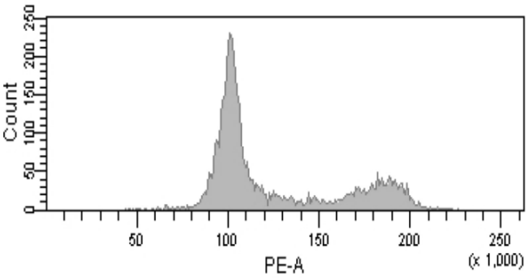

Flubendazole 1µM

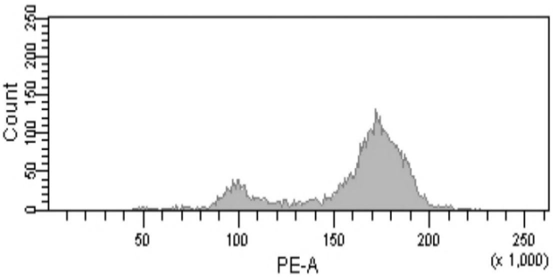

Suppl. Figure S3. Effects of flubendazole on cell cycle distribution in neuroblastoma cells transduced with lentiviral vectors encoding for scrambled, non-targeting shRNA (scr) or shRNA directed against p53 (p53shRNA). Cells were incubated with a flubendazole concentration two times higher that the flubendazole IC_{50} value in the respective cell line for 24h.

Suppl. Figure S4

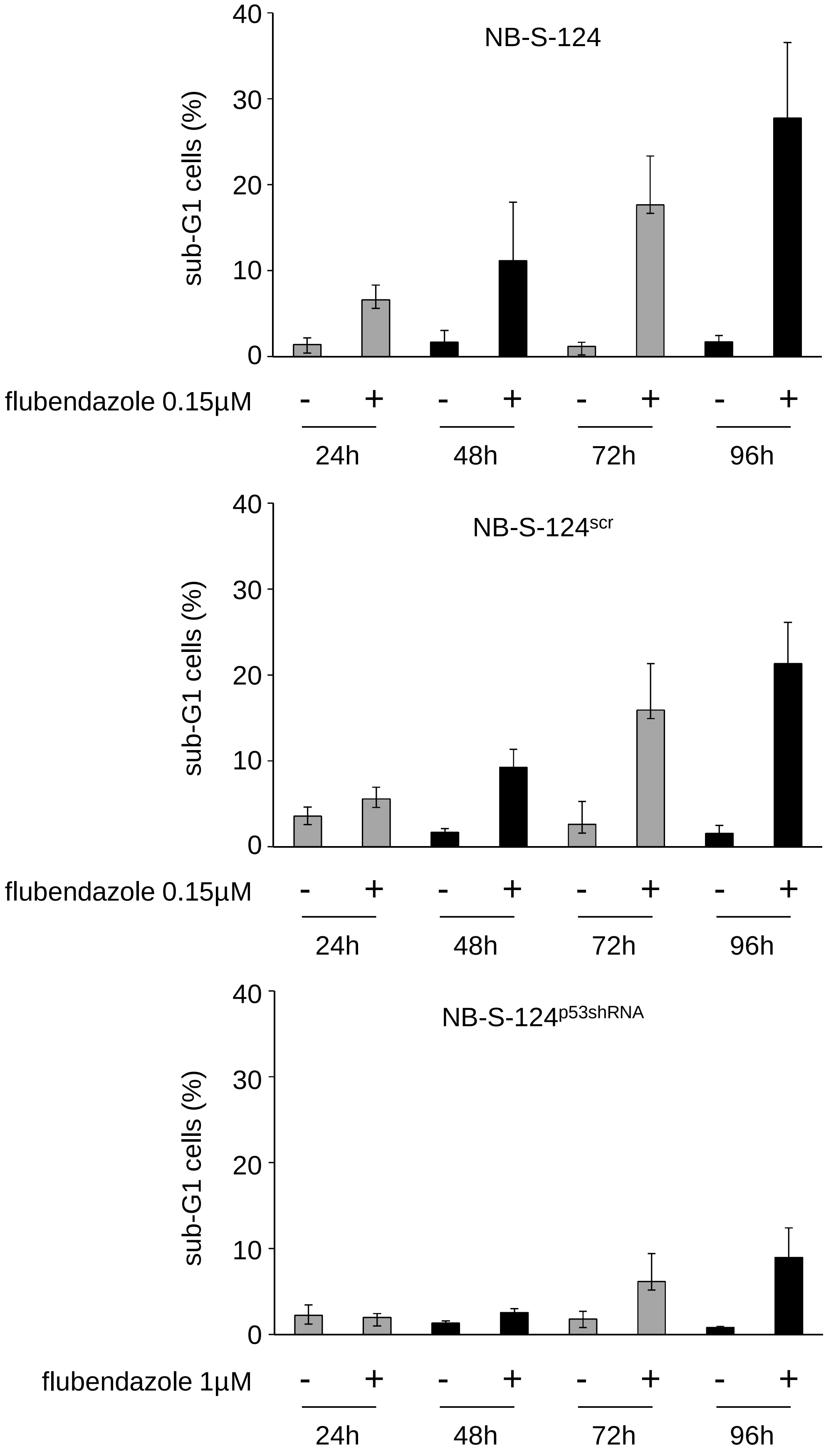

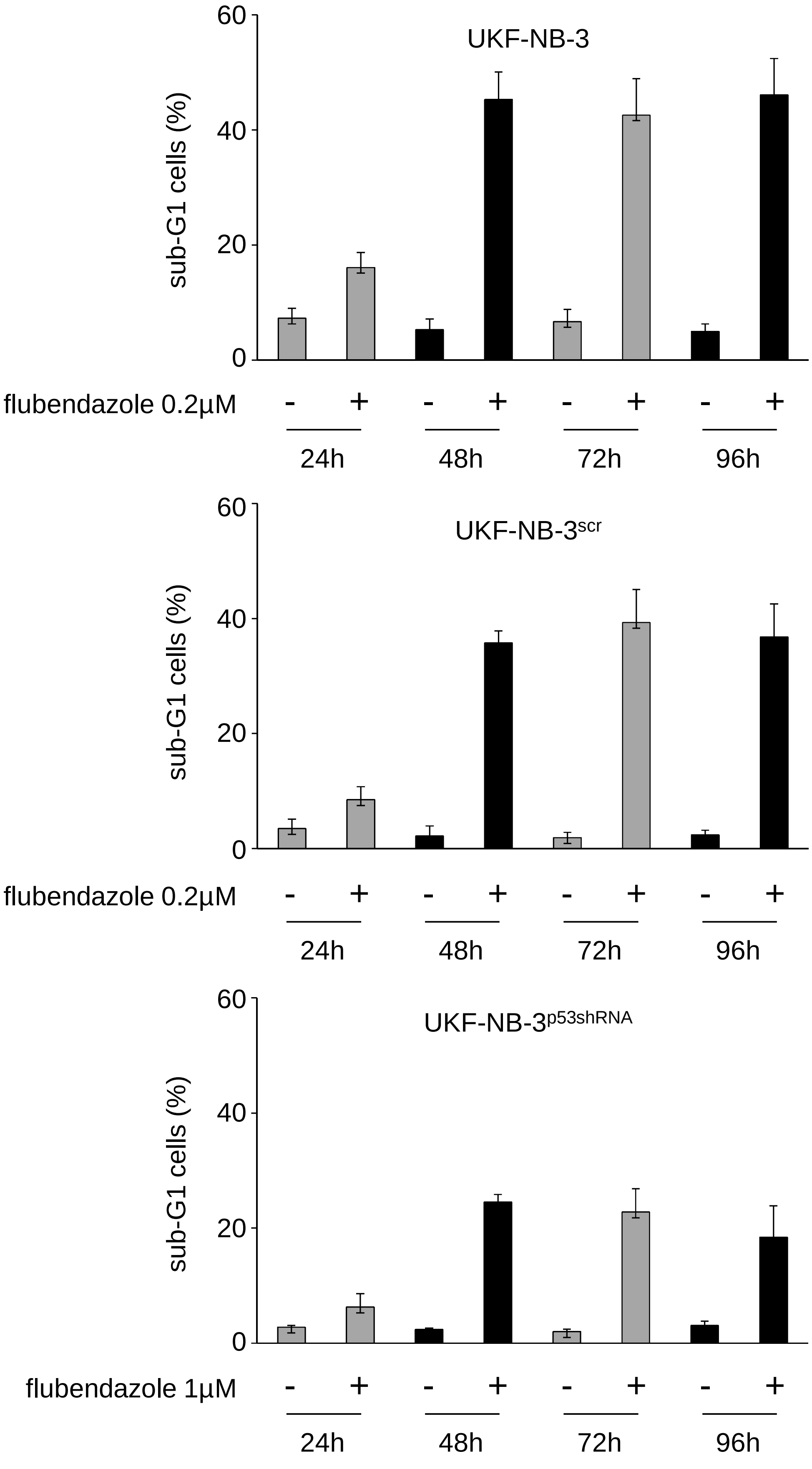
UKF-NB-3
sub-G1 cells (%)
60
40
20
0
flubendazole 0.2µM
- + - + - + - +
24h
48h
72h
96h
UKF-NB-3scr
sub-G1 cells (%)
60
40
20
0
flubendazole 0.2µM
- + - + - + - +
24h
48h
72h
96h
UKF-NB-3p53shRNA
sub-G1 cells (%)
60
40
20
0
flubendazole 1µM
- + - + - + - +
24h
48h
72h
96h

Suppl. Figure S4. Effects of flubendazole on the fraction of sub-G1 cells in p53 wild-type neuroblastoma cells in which p53 was depleted using a lentiviral vector encoding for shRNA directed against p53 (p53shRNA), and neuroblastoma cells transduced with a control vector expressing scrambled, non-targeting shRNA (scr). Flubendazole was used at a concentration two times higher than the flubendazole IC_{50} value in the respective cell lines.

Suppl. Figure S5

A

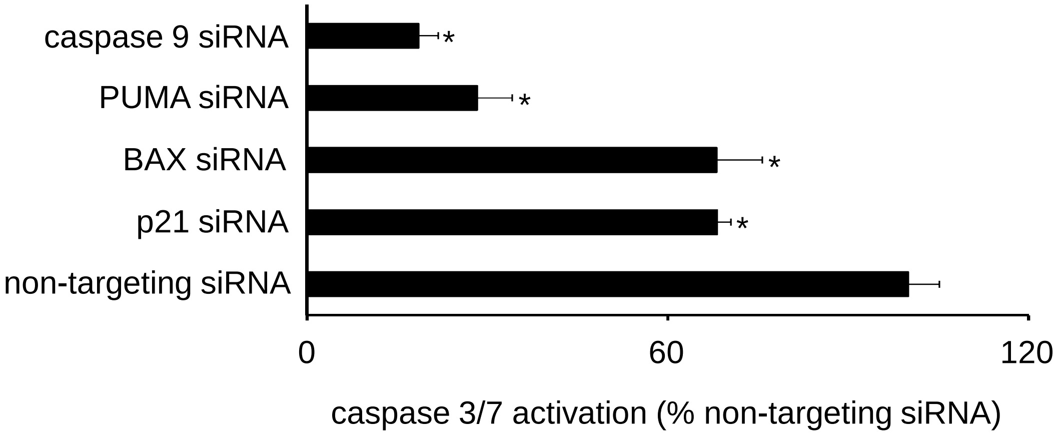

B

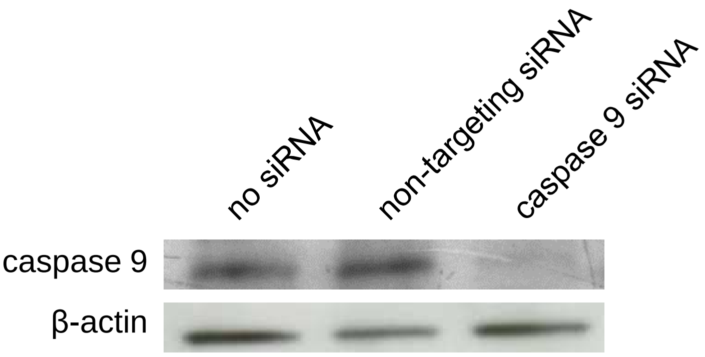

C

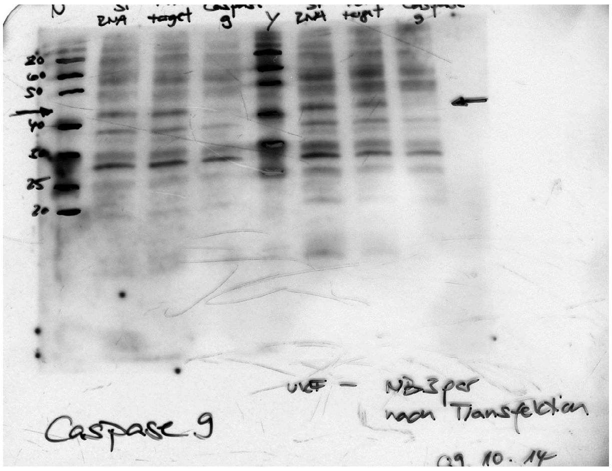

D

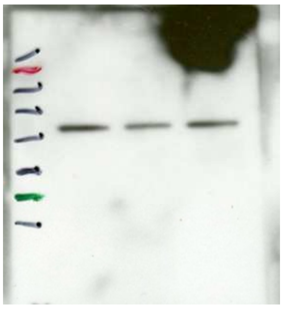

Suppl. Figure S5. A) Effects of siRNA directed against the p53 targets p21, BAX, or PUMA or caspase 9 on caspase 3/7 activation in UKF-NB-3 cells determined after 24h of flubendazole (150nM) treatment. * P < 0.05 relative to non-targeting control siRNA; B) Western blot indicating effects of non-targeting siRNA and siRNA directed against caspase 9 on caspase 9 protein levels in UKF-NB-3 cells (effects of siRNA directed against p21, BAX, and PUMA on the respective protein levels are presented in Figure 4); C) original Western blot indicating siRNA-mediated caspase 9 depletion; arrows indicate the caspase 9 bands. Columns from the left to the right: markers indicating protein size, no siRNA, non-targeting siRNA, caspase 9 siRNA, markers indicating protein size, no siRNA, non-targeting siRNA, caspase 9 siRNA; D) original β-actin Western blot serving as loading control; columns from the left to the right: no siRNA, non-targeting siRNA, caspase 9 siRNA

Suppl. Figure S6

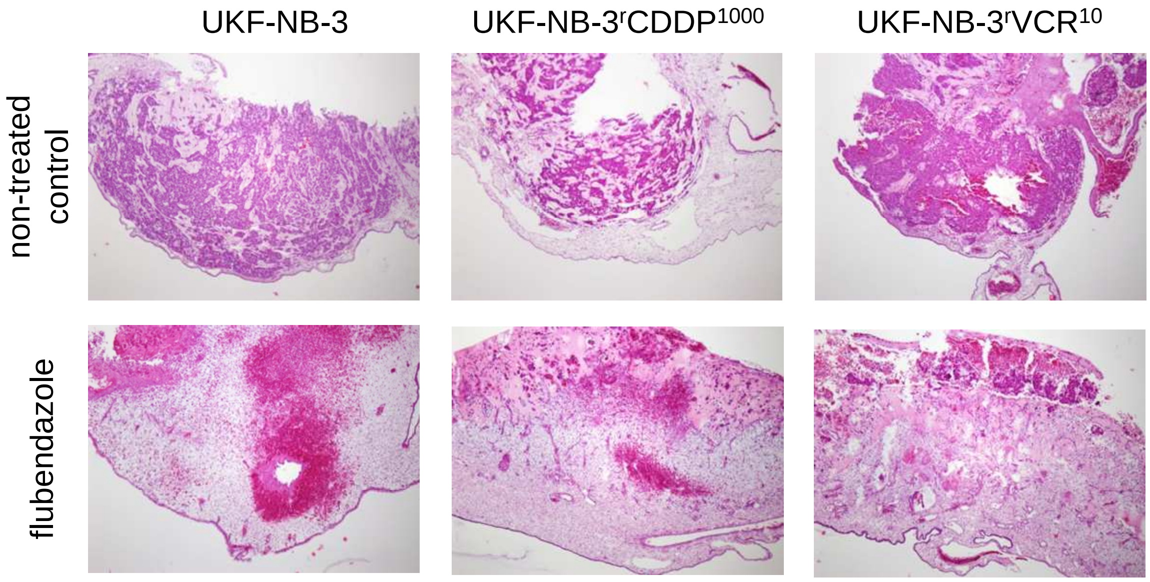

Suppl. Figure S6. Haematoxylin/eosin staining of representative CAMs demonstrating the effects of flubendazole (2µg, applied as (2-hydroxypropyl)-β-cyclodextrin complex in 100µL 0.9% NaCl solution) (original magnification: 4-fold). Flubendazole-treated tumours grow in a less extensive manner and present with large necrotic areas.

Suppl. Figure S7

Original Western blots corresponding to Figure 4A, 4B, and 4C

Figure 4A

p53

Bands from the left to the right

1: UKF-NB-3^{p53shRNA} Nutlin-3 10µM
2: UKF-NB-3^{p53shRNA} non-treated control
3: UKF-NB-3^{p53shRNA} flubendazole 0.3µM
4: UKF-NB-3^{p53shRNA} flubendazole 1.25µM
5: UKF-NB-3^{scr} Nutlin-3 10µM
6: UKF-NB-3^{scr} non-treated control
7: UKF-NB-3^{scr} flubendazole 0.3µM
8: UKF-NB-3^{scr} flubendazole 1.25µM

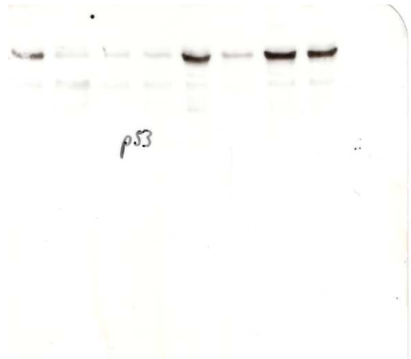

Figure 4A

p53

Bands from the left to the right

1: N/A
2: N/A
3: N/A
4: N/A
5: UKF-NB-3 non-treated control
6: UKF-NB-3 flubendazole 0.3µM
7: UKF-NB-3 flubendazole 1.25µM
8: UKF-NB-3 Nutlin-3 10µM

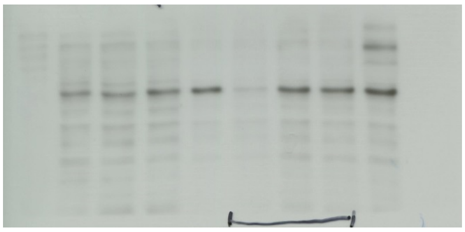

Figure 4A

pp53

Bands from the left to the right

1: UKF-NB-3 non-treated control
2: UKF-NB-3 flubendazole 0.3µM
3: UKF-NB-3 nutlin-3
4: N/A
5: N/A
6: N/A
7: N/A
8: N/A

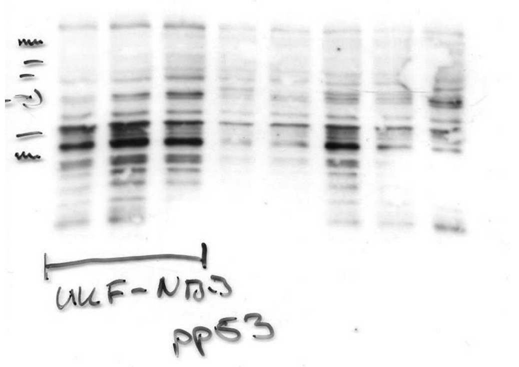

Figure 4A

pp53

Bands from the left to the right

1: UKF-NB-3^{scr} non-treated control
2: UKF-NB-3^{scr} flubendazole 0.08µM
3: UKF-NB-3^{scr} non-treated contro
4: UKF-NB-3^{scr} flubendazole 0.3µM
5: UKF-NB-3^{p53shRNA} non-treated control
6: UKF-NB-3^{p53shRNA} flubendazole 0.08µM
7: UKF-NB-3^{p53shRNA} non-treated control
8: UKF-NB-3^{p53shRNA} flubendazole 0.3µM

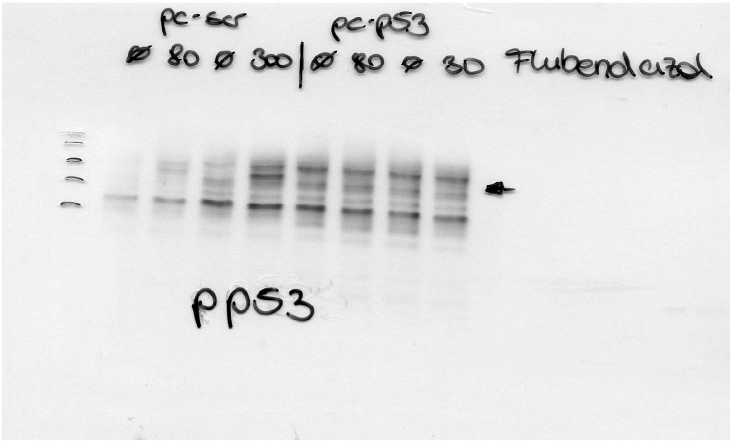

Figure 4A

p21

Bands from the left to the right

1: UKF-NB-3^{p53shRNA} Nutlin-3 10µM
2: UKF-NB-3^{p53shRNA} non-treated control
3: UKF-NB-3^{p53shRNA} flubendazole 0.3µM
4: UKF-NB-3^{p53shRNA} flubendazole 1.25µM
5: UKF-NB-3^{scr} Nutlin-3 10µM
6: UKF-NB-3^{scr} non-treated control
7: UKF-NB-3^{scr} flubendazole 0.3µM
8: UKF-NB-3^{scr} flubendazole 1.25µM

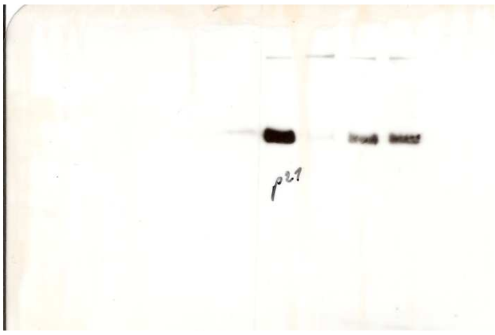

Figure 4A

p21

Bands from the left to the right

1: UKF-NB-3 Nutlin-3 10µM
2: UKF-NB-3 non-treated control
3: UKF-NB-3 flubendazole 0.3µM
4: UKF-NB-3 flubendazole 1.25µM
5: N/A
6: N/A
7: N/A
8: N/A

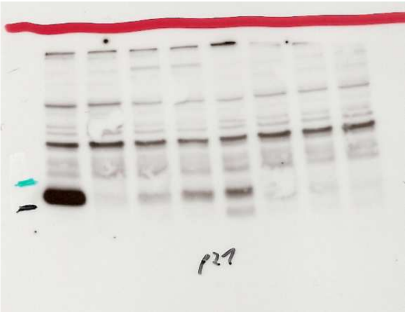

Figure 4A

BAX

- N/A
- N/A
- UKF-NB-3 non-treated control
- UKF-NB-3 0.3µM flubendazole
- N/A
- N/A
- N/A

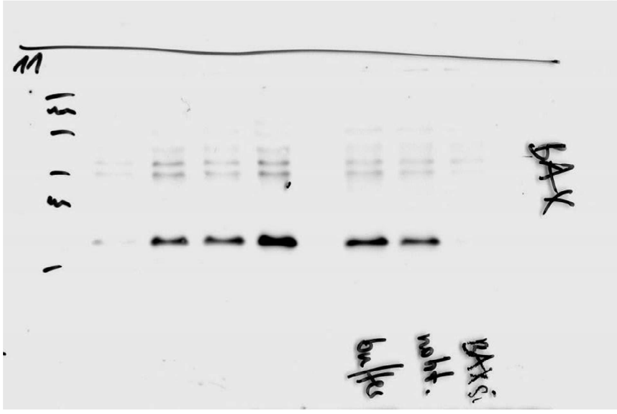

Figure 4A

Bax

Bands from the left to the right

1: UKF-NB-3^{p53shRNA} Nutlin-3 10µM
2: UKF-NB-3^{p53shRNA} non-treated control
3: UKF-NB-3^{p53shRNA} flubendazole 0.3µM
4: UKF-NB-3^{p53shRNA} flubendazole 1.25µM
5: UKF-NB-3^{scr} Nutlin-3 10µM
6: UKF-NB-3^{scr} non-treated control
7: UKF-NB-3^{scr} flubendazole 0.3µM
8: UKF-NB-3^{scr} flubendazole 1.25µM

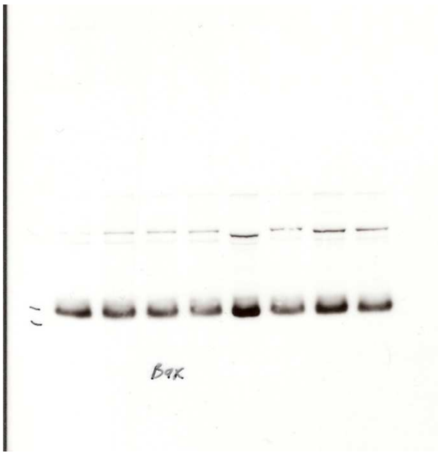

Figure 4A

PUMA

Bands from the left to the right

1: N/A
2: N/A
3: N/A
4: N/A
5: UKF-NB-3 non-treated control
6: UKF-NB-3 flubendazole 0.3µM
7: N/A
8: N/A

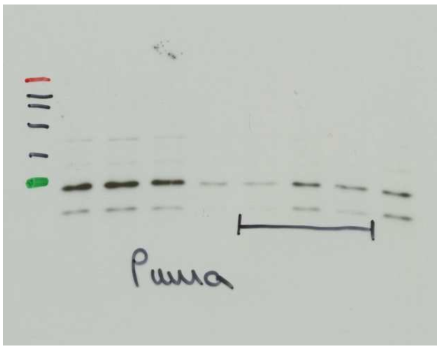

Figure 4A

PUMA

Bands from the left to the right

1: UKF-NB-3^{p53shRNA} Nutlin-3 10µM
2: UKF-NB-3^{p53shRNA} non-treated control
3: UKF-NB-3^{p53shRNA} flubendazole 0.3µM
4: UKF-NB-3^{p53shRNA} flubendazole 1.25µM
5: UKF-NB-3^{scr} Nutlin-3 10µM
6: UKF-NB-3^{scr} non-treated control
7: UKF-NB-3^{scr} flubendazole 0.3µM
8: UKF-NB-3^{scr} flubendazole 1.25µM

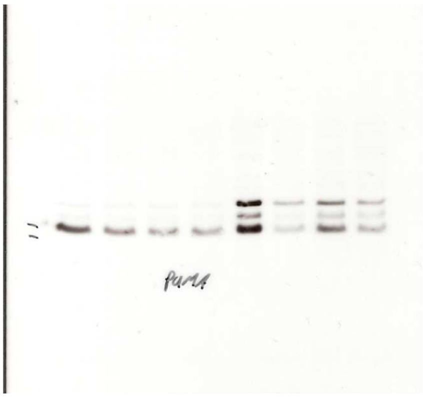

Figure 4B

Caspase 3

Bands from the left to the right

1: N/A
2: N/A
3: UKF-NB-3^{scr} non-treated control
4: UKF-NB-3^{scr} flubendazole 0.3µM
5: UKF-NB-3^{p53shRNA} non-treated control
6: UKF-NB-3^{p53shRNA} flubendazole 0.3µM
7: N/A
8: N/A

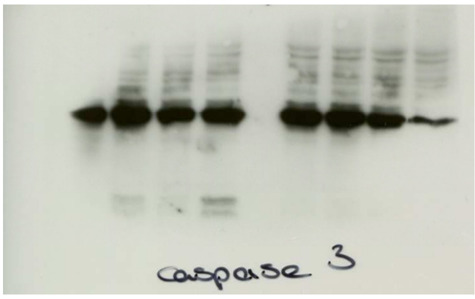

Figure 4B

Caspase 3

Bands from the left to the right

1: UKF-NB-3 non-treated
2: UKF-NB-3 flubendazole 0.3µM
3: N/A
4: N/A
5: N/A
6: N/A

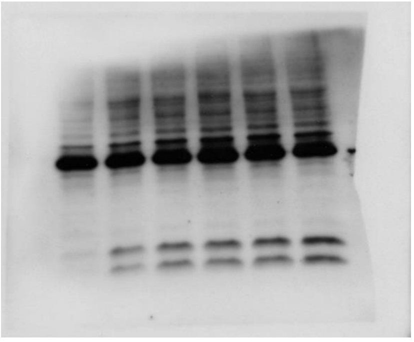

Figure 4B

PARP

Bands from the left to the right

1: UKF-NB-3$^{\text{p53shRNA}}$ Nutlin-3 10µM
2: UKF-NB-3$^{\text{p53shRNA}}$ non-treated control
3: UKF-NB-3$^{\text{p53shRNA}}$ flubendazole 0.3µM
4: UKF-NB-3$^{\text{p53shRNA}}$ flubendazole 1.25µM
5: UKF-NB-3^{scr} Nutlin-3 10µM
6: UKF-NB-3^{scr} non-treated control
7: UKF-NB-3^{scr} flubendazole 0.3µM
8: UKF-NB-3^{scr} flubendazole 1.25µM

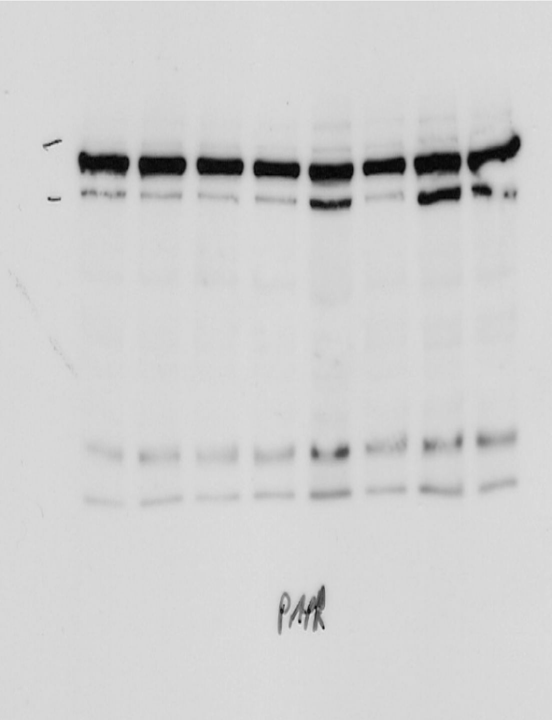

Figure 4B

PARP

Bands from the left to the right

1: UKF-NB-3 Nutlin-3 10µM
2: UKF-NB-3 non-treated control
3: UKF-NB-3 flubendazole 0.3µM
4: UKF-NB-3 flubendazole 1.25µM
5: N/A
6: N/A
7: N/A
8: N/A

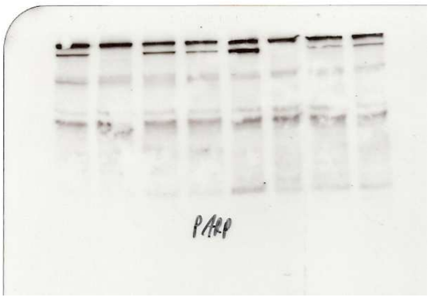

Figure 4B

β-actin

Bands from the left to the right

1: UKF-NB-3$^{\text{p53shRNA}}$ Nutlin-3 10µM
2: UKF-NB-3$^{\text{p53shRNA}}$ non-treated control
3: UKF-NB-3$^{\text{p53shRNA}}$ flubendazole 0.3µM
4: UKF-NB-3$^{\text{p53shRNA}}$ flubendazole 1.25µM
5: UKF-NB-3^{scr} Nutlin-3 10µM
6: UKF-NB-3^{scr} non-treated control
7: UKF-NB-3^{scr} flubendazole 0.3µM
8: UKF-NB-3^{scr} flubendazole 1.25µM

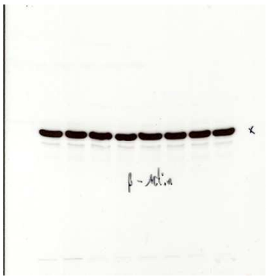

Figure 4B

β-actin

Bands from the left to the right

1: N/A
2: N/A
3: N/A
4: N/A
5: UKF-NB-3 Nutlin-3 10µM
6: UKF-NB-3 non-treated control
7: UKF-NB-3 flubendazole 0.3µM
8: UKF-NB-3 flubendazole 1.25µM

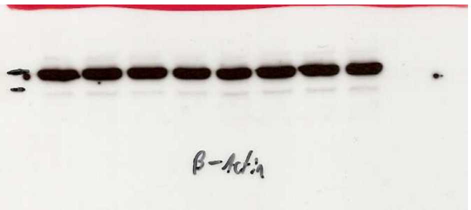

Figure 4C

p21

from the left to the right:

- no siRNA
- non-targeting siRNA
- p21 siRNA

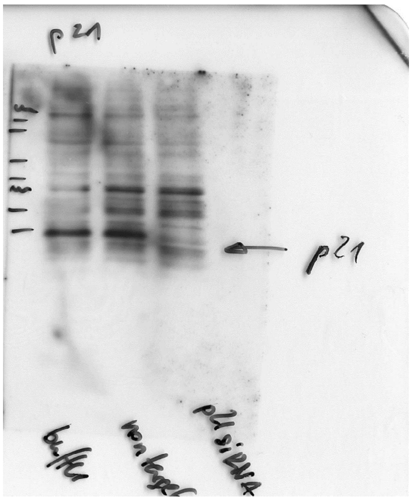

Figure 4C

BAX

- N/A
- N/A
- N/A
- N/A
- no siRNA
- non-targeting siRNA
- BAX siRNA

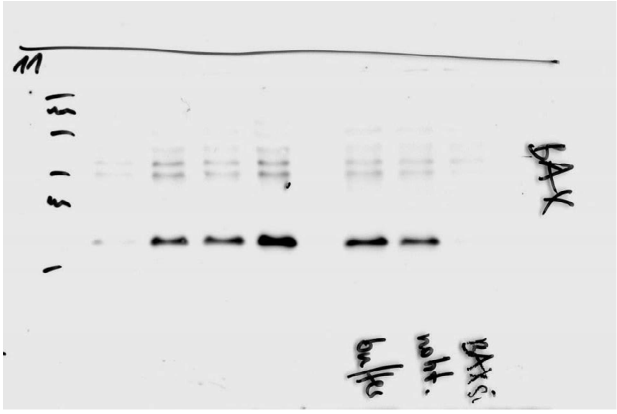

Figure 4C

PUMA

from the left to the right:

- no siRNA
- non-targeting siRNA
- PUMA siRNA

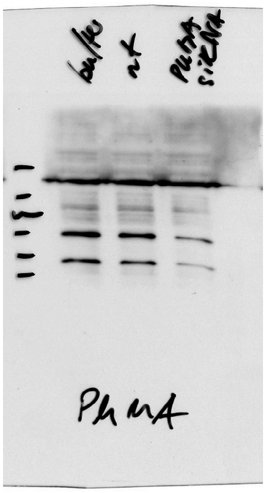

Figure 4C

β-actin

from the left to the right:

p21

- no siRNA
- non-targeting siRNA
- p21 siRNA

BAX

- no siRNA
- non-targeting siRNA
- BAX siRNA

PUMA

- no siRNA
- non-targeting siRNA
- PUMA siRNA

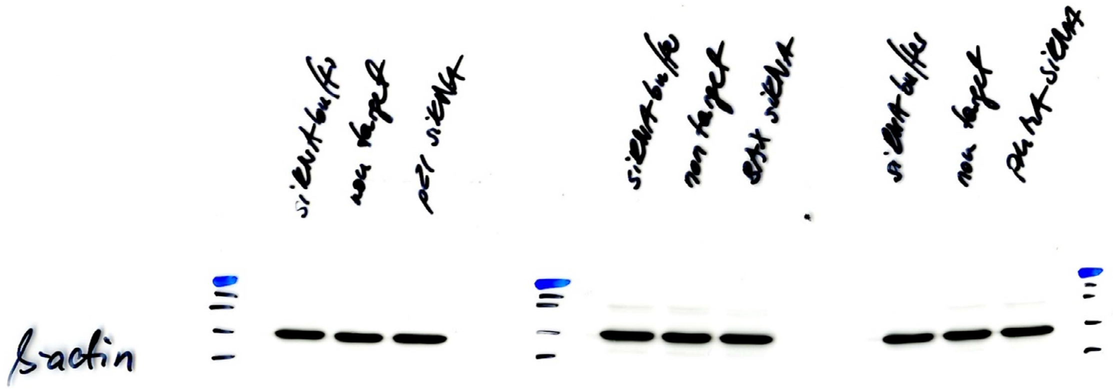

Suppl. Table S1. Flubendazole concentrations that inhibit the viability of the investigated cell lines by 50% (IC_{50}) or 90% (IC_{90}) as indicated by MTT assay after a five day incubation period.

**Cell line**	**source/ medium**	**IC_{50}/IC_{90} (nM) flubendazole**
**Breast cancer**		
BT20	ATCC[1]/MEM[2]	1287 ± 100 / > 5000
B-474	DSMZ/RPMI 1640 + insulin	2519 ± 493 / > 5000
BT-549	CLS/DMEM	168.9 ± 5.6 / 1016 ± 31
CAL-51	DMSZ/ DMEM	1010 ± 125 / > 5000
CAL-148	DSMZ/ DMEM	190.2 ± 27.6 / 355.4 ± 40.9
DU-4475	DSMZ/ RPMI 1640	141.6 ± 18.4 / 296.3 ± 31.6
HCC38	ATCC/RPMI 1640	234.6 ± 21.8 / > 5000
HCC70	ATCC/RPMI 1640	> 5000 / > 5000
HCC-1143	DSMZ/ RPMI 1640	> 5000 / > 5000
HCC1806	ATCC/ RPMI 1640	159.0 ± 4.6 / 603.4 ± 75.2
HCC1937	ATCC/ RPMI 1640	> 5,000 / > 5000
HDQ-P1	DSMZ/ DMEM	1119 ± 105 / > 5000
Hs 578T	ECACC /DMEM	243.8 ± 33.9 / 362.6 ± 46.8
MCF-7	DSMZ/RPMI 1640 + insulin	> 5000 / > 5000
MDA-MB-231	CLS/ DMEM Ham's F12	513.6 ± 76.3 / > 5000
MDA-MB-436	CLS/ DMEM Ham's F12	660.0 ± 75.3 / > 5000
MDA-MB-453	DSMZ/Leibovitz´s L15	237.7 ± 29.6 / > 5000
MDA-MB-468	ATCC/Leibovitz´s L15	481.0 ± 72.5 / > 5000
SK-BR-3	ATCC/ IMDM	354.2 ± 41.0 / > 5000
SUM149PT	Asterand/ DMEM Ham's F12 + insulin and hydrocortisone	332.2 ± 29.2 / 500.2 ± 61.4
SUM159PT	Asterand/ DMEM Ham's F12 + insulin and hydrocortisone	642.4 ± 52.3 / 1518 ± 196
**Cervix cancer**		
C-4 I	ATCC/ Waymouth´s MB 752/1	280.8 ± 25.7 / > 5000
CaSki	CLS/ RPMI 1640	326.1 ± 40.6 / > 5000
CERV-186	CLS/ DMEM Ham´s F12	219.7 ± 29.6 / 351.3 ± 39.7
CERV-196	CLS/ DMEM Ham´s F12	784.8 ± 95.2 / > 5000
CERV-215	CLS/ Ham´s F12	538.5 ± 44.4 / > 5000
HeLa	ATCC/MEM	360.2 ± 33.5 / 1374 ± 101
ME-180	JCRB/MEM	650.5 ± 113.8 / 3128 ± 584
MS751	ATCC/ MEM	98.6 ± 12.5 / 301.8 ± 35.1

SISO	DSMZ/ RPMI 1640	243.6 ± 29.3 / 825.4 ± 99.4
SKG-IIIa	JCRB/Ham´s F12	3042 ± 594 / > 5000
**Colorectal cancer**		
Caco-2	DSMZ/ IMDM	1025 ± 193 / > 5000
CL-11	DSMZ/ DMEM Ham´s F12	595.0 ± 68.4 / > 5000
CL-14	DSMZ/DMEM Ham´s F12	1229.2 ± 151.6 / > 5000
CL-34	DSMZ/ DMEM Ham´s F12	179.2 ± 20.8 / 366.4 ± 45.1
DLD-1	DSMZ/ RPMI 1640	365.0 ± 46.5 / > 5000
HCT-15	DSMZ/ RPMI 1640	213.3 ± 18.2 / > 5000
HT-29	DSMZ/ McCoy´s 5a	272.7 ± 10.1 / > 5000
LoVo	ICLC/RPMI 1640	352.6 ± 21.0 / > 5000
RKO	ATCC/ MEM	252.1 ± 9.4 / 2739 ± 411
SW48	ATCC/Leibovitz´s L15	335.8 ± 49.6 / > 5000
SW-403	DSMZ/ DMEM	240.1 ± 28.3 / > 5000
SW480	ATCC/Leibovitz´s L15	367.6 ± 44.9 / > 5000
SW620	ATCC/Leibovitz´s L15	269.3 ± 34.8 / > 5000
**Ewing’s sarcoma**		
A-673	ATCC/DMEM	399.5 ± 15.3 / 469.5 ± 25.8
CADO-ES-1	DSMZ/ RPMI 1640	475.2 ± 23.9 / > 5000
MHH-ES-1	DSMZ/ RPMI 1640	130.7 ± 6.9 / 204.6 ± 22.1
RD-ES	DSMZ/ RPMI 1640	473.9 ± 18.0 / 2579 ± 312
Rh1	Houghton/ IMDM	283.1 ± 12.3 / 2884 ± 351
SK-ES-1	DSMZ/ McCoy´s 5a	130.5 ± 12.1 / 270.5 ± 23.6
TC-71	DSMZ/ IMDM	408.9 ± 25.2 / 597.3 ± 37.9
**Gastric cancer**		
23132/87	DSMZ/ RPMI 1640	709.5 ± 98.1 /> 5000
AGS	CLS/ DMEM	223.8 ± 24.5 / > 5000
CLS-145	CLS/ Ham´s F12	2494 ± 286 / > 5000
HGC-27	CLS/ DMEM Ham´s F12	248.5 ± 29.2 / > 5000
KATO-III	CLS/ Ham´s F12	349.6 ± 39.4 / > 5000
MKN1	JCRB/RPMI 1640	> 5000 / > 5000
MKN45	JCRB/RPMI 1640	605.4 ± 103.8 / > 5000
NUGC-4	JCRB/RPMI 1640	> 5000 /> 5000
SK-GT-2	DSMZ/ MEM	510.2 ± 75.3 / 2881 ± 301
**Glioma**		
A-172	ATCC/DMEM	309.8 ± 25.1 / 2098 ± 177
D247	Naumann/ IMDM	301.8 ± 38.2 / > 5000
G62	von Laer/ IMDM	355.3 ± 13.6 / > 5000
GMS-10	DSMZ/ DMEM	582.3 ± 20.7 / > 5000
GOS-3	DSMZ/ DMEM	432.4 ± 68.6 / > 5000
LN-18	ATCC/ DMEM	336.3 ± 37.9 / 604.6 ± 73.1
LN-229	ATCC/ DMEM	363.0 ± 31.9 / > 5000

LN-405	DSMZ/ DMEM	> 5,000 / > 5000
MZ18	Edinger Institute/ IMDM	1031 ± 127 / > 5000
T98G	ATCC/ IMDM	302.4 ± 33.8 / > 5000
TU132	Naumann/ IMDM	> 5000 / > 5000
TU140	Naumann/ IMDM	342.1 ± 39.0 / > 5000
U-138 MG	ATCC/MEM	> 5000 / > 5000
U138	Naumann/ IMDM	338.2 ± 39.6 / > 5000
U-251 MG	CLS / MEM	> 5000 / > 5000
U343	CLS /MEM	304.7 ± 4.1 / > 5000
U373MG	ATCC/ IMDM	374.0 ± 40.4 / > 5000
U-87 MG	ATCC/MEM	> 5000 / > 5000
**Head and neck cancer**		
CAL 27	ATCC/DMEM	180.9 ± 11.8 / > 5000
CAL-33	DSMZ/ DMEM	86.3 ± 12.6 / 639.0 ± 89.5
CLS-354	CLS/ DMEM Ham´s F12	82.0 ± 7.6 / 262.6 ± 29.2
SCC-4	ATCC/DMEM F12 + hydrocortisone	378.8 ± 23.6 / > 5000
SCC25	DSMZ/ DMEM Ham's F12	359.2 ± 28.3 / 655.7 ± 80.8
**Hepatocellular carcinoma**		
HEP-3B	DSMZ/ MEM	436.7 ± 54.7 / > 5000
HEP-G2	DSMZ/RPMI 1640	362.7 ± 19.3 / > 5000
HLE	JCRB/DMEM	1605 ± 326 / > 5000
HLF	JCRB/DMEM	2619 ± 455 / > 5000
HUH-6 Clone 5	JCRB/DMEM	> 5000 / > 5000
HuH-7	JCRB /DMEM	> 5000 / > 5000
PLC-PRF-5	CLS/ DMEM	459.0 ± 69.4 / > 5000
SK-HEP-1	CLS/ RPMI 1640	> 5000 / > 5000
SNU-182	ATCC/ RPMI 1640	395.3 ± 42.7 / 882.6 ± 92.3
SNU-449	ATCC/ RPMI 1640	345.9 ± 38.3 / > 5000
**Leukaemia/lymphoma**		
C8166	NIBSC/RPMI 1640	51.4 ± 6.7 / 101.7 ± 12.5
CCRF-CEM	ATCC/ RPMI 1640	221.7 ± 34.9 / 495.5 ± 50.3
CTV-1	DSMZ/ RPMI 1640	67.0 ± 13.6 / 112.7 ± 13.6
EM-2	DSMZ/ RPMI 1640	167.1 ± 21.8 / 582.5 ± 62.6
EM-3	DSMZ/ RPMI 1640	524.2 ± 67.4 / 4988 ± 322
H9	ATCC/ RPMI 1640	398.9 ± 25.2 / > 5000
HDLM2	DSMZ/ RPMI 1640	204.9 ± 17.6 / 968.2 ± 121.5
HEL	DSMZ/ RPMI 1640	358.2 ± 27.5 / 905.8 ± 98.3
HL60	ATCC/ IMDM	111.9 ± 18.1 / 236.2 ± 22.7
HuT 78	ATCC/ IMDM	227.7 ± 16.3 / 343.5 ± 29.4
JJHan	ATCC/ IMDM	336.2 ± 39.6 / 962.8 ± 105.1
JK-1	DSMZ/ RPMI 1640	73.7 ± 16.0 / 231.6 ± 44.7

JURKAT	DSMZ/ RPMI 1640	123.3 ± 10.5 / 201.8 ± 10.3
K-562	ATCC/ IMDM	98.0 ± 8.5 / 347.2 ± 48.6
KARPAS-231	DSMZ/ RPMI 1640	230.5 ± 27.1/ 459.2 ± 50.6
KASUMI-2	DSMZ/ RPMI 1640	272.4 ± 16.8 / 567.3 ± 30.8
KCL-22	DSMZ/ RPMI 1640	345.3 ± 39.3 / 571.6 ± 71.8
KE-37	DSMZ/ RPMI 1640	285.6 ± 39.2 / 533.5 ± 78.1
KG-1	DSMZ/ RPMI 1640	80.4 ± 11.7 / 246.2 ± 47.9
KM-H2	DSMZ/ RPMI 1640	153.9 ± 13.6 / 3184 ± 227
KU-812	DSMZ/ RPMI 1640	105.0 ± 12.3 / 229.2 ± 34.1
KYO-1	DSMZ/ RPMI 1640	247.9 ± 29.9 / 291.4 ± 22.8
L-1236	DSMZ/ RPMI 1640	80.0 ± 14.9 / > 5000
L-428	DSMZ/ RPMI 1640	178.9 ± 23.2 / 1229 ± 151
L-540	DSMZ/ RPMI 1640	79.2 ± 8.9 / 321.3 ± 36.6
LAMA-84	DSMZ/ RPMI 1640	170.3 ± 21.4 / 464.7 ± 63.1
LOUCY	DSMZ/ RPMI 1640	198.5 ± 27.1 / 553.2 ± 72.9
MEG-01	DSMZ/ RPMI 1640	296.4 ± 30.0 / 588.5 ± 75.8
MHH-CALL-4	DSMZ/ RPMI 1640	229.6 ± 46.2 / > 5000
MN-60	DSMZ/ Ham´s F10	314.4 ± 39.3 / 463.3 ± 55.8
MOLM-6	DSMZ/ RPMI 1640	53.7 ± 14.6 / 119.8 ± 13.9
MOLM-13	DSMZ/ RPMI 1640	175.9 ± 6.5 / 406.9 ± 43.7
MOLT-4	DSMZ/ RPMI 1640	180.3 ± 21.7 / 424.7 ± 33.4
MT2	ECACC/ RPMI 1640	94.7 ± 9.1 / 554.6 ± 63.8
MT4	ECACC/RPMI 1640	110.5 ± 27.6 / 120.0 ± 31.5
MV4-11	DSMZ/ RPMI 1640	498.2 ± 57.4 / 887.0 ± 115.8
OCI-AML-2	DSMZ/ alpha-MEM	337.5 ± 58.6 / 585.1 ± 46.9
OCI-AML-3	DSMZ/ alpha-MEM	150.1 ± 21.8 / 290.2 ± 30.4
OCI-AML-5	DSMZ/ alpha-MEM + GM-CSF	445.1 ± 80.8 / 790.2 ± 60.8
P12-ICHIKAWA	DSMZ/ RPMI 1640	> 5000 / > 5000
PL-21	DSMZ/ RPMI 1640	134.7 ± 16.6 / 218.9 ± 23.0
ROS-50	DSMZ/ RPMI 1640	270.0 ± 36.4 / 4672 ± 508
SEM	DSMZ/ IMDM	313.0 ± 39.4 / 531.8 ± 61.5
SIG-M5	DSMZ/ IMDM	126.3 ± 8.7 / 261.5 ± 28.3
SUP-T11	DSMZ/ RPMI 1640	141.8 ± 20.6 / 251.6 ± 38.7
TANOUE	DSMZ/ RPMI 1640	296.5 ± 48.1 / > 5000
THP-1	ATCC / RPMI 1640 + 2-mercaptoethanol	149.8 ± 30.0 / 337.6 ± 62.2
U-937	DSMZ/ RPMI 1640	84.4 ± 9.5 / 167.2 ± 12.5
UT-7	DSMZ/ alpha-MEM	113.0 ± 16.8 / 752.0 ± 86.9
**Lung cancer**		
A549	ATCC/ MEM	372.5 ± 47.9 / > 5000
CAL-12T	DSMZ/DMEM	328.4 ± 37.8 / 2946 ± 393
CaLu-1	CLS/MEM	241.4 ± 27.3 / > 5000
Calu-6	CLS/ RPMI 1640	106.6 ± 12.2 / 2473 ± 205
DMS-79	CLS/ RPMI 1640	> 5000 / > 5000
H-1339	DSMZ/RPMI 1640	261.7 ± 32.8 / > 5000
H2228	ATCC/ RPMI 1640	> 5000 / > 5000

HCC-15	DSMZ/ RPMI 1640	216.4 ± 34.6 / 293.4 ± 30.8
HCC-44	DSMZ/ RPMI 1640	208.8 ± 20.4 / 485.1 ± 40.7
HCC-366	DSMZ/RPMI 1640	331.2 ± 46.9 / > 5000
HCC-827	DSMZ/ RPMI 1640	225.1 ± 25.6 / > 5000
HCC4006	ATCC/ RPMI 1640	286.6 ± 41.9 / > 5000
LCLC-97TM1	DSMZ/RPMI 1640	62.6 ± 8.0 / 216.4 ± 28.9
NCI-H69	CLS/ RPMI 1640	409.8 ± 60.8 / 676.4 ± 82.2
NCI-H82	CLS/ RPMI 1640	198.1 ± 25.4 / 1337 ± 144
NCI-H146	CLS/RPMI 1640	> 5000 / > 5000
NCI-H209	CLS/IMDM	291.5 ± 8.5 / 564.3 ± 38.3
PC-9	ECACC/RPMI 1640	126.0 ± 3.6 / 301.6 ± 5.7
SCLC-22H	CLS/DMEM	190.9 ± 3.1 / > 5000
SK-MES-1	CLS/DMEM	138.8 ± 15.2 / > 5000
**Medulloblastoma**		
CHLA-01-MED	ATCC/DMEM F12 + EGF, bFGF, B-27 supplement	286.4 ± 20.0 / > 5000
D283 Med	ATCC/MEM	121.6 ± 9.3 / 246.8 ± 45.0
D341 Med	ATCC/MEM	2453 ± 287 / > 5000
Daoy	ATCC/MEM	176.6 ± 13.1 / 291.7 ± 22.6
HD-MB03	Witt/ IMDM	124.6 ± 15.6 / 374.8 ± 20.3
MHH-Med-1	Pietsch/ IMDM	445.0 ± 32.2 / 1965 ± 204
ONS-76	Edinger Institute/ IMDM	> 5000 / > 5000
UW228	Edinger Institute/ IMDM	520.0 ± 59.1 / > 5000
**Melanoma**		
Colo-679	DSMZ/ IMDM	477.2 ± 33.4 / > 5000
IGR-1	DSMZ/ IMDM	232.6 ± 25.8 / > 5000
IGR-39	DSMZ/ IMDM	317.4 ± 14.2 / 3135 ± 442
IPC-298	DSMZ/ IMDM	453.6 ± 59.7 / > 5000
MelHO	DSMZ/ IMDM	422.3 ± 30.1 / > 5000
MelJuso	DSMZ/ IMDM	1849 ± 279 / > 5000
MeWo	ATCC/ IMDM	381.5 ± 39.2 / > 5000
RPMI-7951	DSMZ/DMEM	1112 ± 203 / > 5000
RVH-421	DSMZ/ IMDM	614.1 ± 48.6 / > 5000
SK-Mel-3	DSMZ/ IMDM	256.7 ± 30.3 / > 5000
SK-Mel-30	DSMZ/ IMDM	490.8 ± 20.6 / > 5000
SK-Mel-31	ATCC/ IMDM	399.2 ± 47.1 / > 5000
WM164	Wistar Institute/ IMDM	284.2 ± 34.9 / > 5000
**Mesothelioma**		
DM-3	DSMZ/ DMEM	2102 ± 249 / > 5000
JL-1	DSMZ/ DMEM	537.5 ± 61.4 / > 5000
MSTO-211H	DSMZ/ RPMI 1640	392.8 ± 66.1 / > 5000
RS-5	DSMZ/NCTC-109	1910 ± 244 / > 5000
ZL5	ECACC/DMEM Ham´s F12	155.6 ± 16.4 / 1892 ± 210

ZL55	ECACC / DMEM Ham´s F12	151.4 ± 16.9 / 513.0 ± 76.6
**Multiple myeloma**		
EJM	DSMZ/ IMDM	358.6 ± 61.9 / 857.5 ± 250.6
JIM1	ECACC/Fischer´s medium + hydrocortisone (Dexter culture medium)	162.5 ± 20.7 / 337.4 ± 40.8
KARPAS 417	ECACC/RPMI 1640	216.0 ± 34.8 / 462.5 ± 59.3
KARPAS-929	ECACC/RPMI 1640	258.6 ± 45.1 / 309.2 ± 34.7
KMS-12-BM	DSMZ/ RPMI 1640	189.4 ± 15.3 / 474.5 ± 52.1
KMS-12-PE	DSMZ/ RPMI 1640	96.1 ± 7.3 / 181.3 ± 35.2
MOLP-8	DSMZ/ RPMI 1640	174.4 ± 42.1 / 408.7 ± 45.0
OPM-2	DSMZ/ RPMI 1640	289.0 ± 10.5 / 411.9 ± 31.0
RPMI-8226	CLS/ RPMI 1640	96.2 ± 23.6 / 384.3 ± 72.8
U-266	DSMZ/RPMI 1640	161.9 ± 13.2 / 382.7 ± 30.4
**Neuroblastoma**		
Be(2)C	ATCC/ IMDM	114.5 ± 19.7 / 573.0 ± 66.2
CHP-134	DSMZ/ RPMI 1640	59.8 ± 6.7 / 76.6 ± 8.1
GI-CA-N	ICLC/ IMDM	181.7 ± 22.3 / 275.1 ± 32.3
GI-ME-N	ICLC/ RPMI 1640	97.9 ± 14.7 / 614.6 ± 81.3
GOTO	JCRB/RPMI 1640	461.3 ± 72.8 / 1985 ± 406
IMR-5	Eggert/ IMDM	125.9 ± 7.0 / 266.3 ± 23.7
IMR-32	ATCC/ IMDM	97.2 ± 42.8 / 226.0 ± 23.1
Kelly	ECACC / RPMI 1640	285.1 ± 31.8 / 805.4 ± 96.3
LAN-1	DSMZ/ IMDM	94.7 ± 18.4 / 422.9 ± 57.0
LAN-2	DSMZ/ RPMI 1640	75.3 ± 10.6 / 212.5 ± 25.9
LAN-5	ICLC/ IMDM	249.0 ± 33.7 / 674.8 ± 75.5
LAN-6	DSMZ / DMEM	228.2 ± 39.4 / 666.3 ± 99.5
LS	DSMZ/ RPMI 1640	312.8 ± 42.4 / 368.9 ± 45.7
MHH-NB-11	DSMZ/ RPMI 1640	85.1 ± 16.3 / 137.1 ± 22.5
NB69	ECACC/ RPMI 1640	348.5 ± 45.1 / 1155 ± 137
NB-S-124	Westermann/ IMDM	75.2 ± 20.2 / 159.5 ± 8.4
NBL-S	DSMZ/IMDM	293.9 ± 40.2 / 607.5 ± 86.1
NGP	DSMZ/ IMDM	303.7 ± 45.3 / 1781 ± 451
NLF	Eggert/ IMDM	141.0 ± 15.6 / 261.9 ± 77.0
NMB	DSMZ/DMEM	53.0 ± 6.5 / 306.6 ± 33.9
SHEP	Eggert/ IMDM	225.8 ± 8.3 / 426.1 ± 12.9
SH-SY5Y	ATCC/ MEM F12	166.8 ± 14.6 / 479.7 ± 58.6
SIMA	DSMZ/ RPMI 1640	45.7 ± 6.4 / 119.0 ± 25.8
SK-N-AS	ATCC/ DMEM	159.3 ± 38.8 / 352.3 ± 84.0
SK-N-F1	ECACC/DMEM	168.2 ± 18.0 / > 5000
SK-N-SH	ATCC/ MEM	133.7 ± 20.9 / 341.6 ± 39.0
UKF-NB-1	Cinatl/ IMDM	243.6 ± 29.3 / 567.1 ± 72.8
UKF-NB-2	Cinatl/ IMDM	128.0 ± 22.3 / 283.4 ± 35.1
UKF-NB-3	Cinatl/ IMDM	48.0 ± 7.9 / 102.3 ± 12.7
UKF-NB-4	Cinatl/ IMDM	134.9 ± 33.4 / 276.8 ± 22.0

UKF-NB-5	Cinatl/ IMDM	113.8 ± 7.5 / 231.6 ± 19.8
UKF-NB-6	Cinatl/ IMDM	207.3 ± 19.3 / 335.6 ± 49.9
**Oesophageal cancer**		
COLO-680N	CLS/ RPMI 1640	180.0 ± 65.5 / > 5000
KYSE-30	DSMZ/RPMI 1640 + Ham´s F12	180.7 ± 25.4 / > 5000
KYSE-70	DSMZ/RPMI 1640	650.0 ± 52.3 / > 5000
KYSE-140	DSMZ/ RPMI 1640	294.8 ± 11.8 / > 5000
KYSE-450	DSMZ/ RPMI 1640 Ham´s F12	139.4 ± 22.5 / 1451 ± 137
T.T	JCRB/DMEM Ham´s F12	4248 ± 669 / > 5000
**Osteosarcoma**		
143B	ATCC/MEM	316.3 ± 22.6 / > 5000
CAL-72	DSMZ/DMEM	251.0 ± 33.5 / > 5000
HOS	ICLC/DMEM	460.8 ± 59.0 / > 5000
MG-63	ICLC/DMEM	436.3 ± 51.8 / > 5000
NY	JCRB/ MEM	369.6 ± 71.5 / 2647 ± 538
SAOS-2	DSMZ/McCoy´s 5a	271.8 ± 24.7 / > 5000
SJSA-1	ATCC/RPMI 1640	661.7 ± 98.3 / > 5000
UKF-OS-1	Cinatl/IMDM	142.4 ± 12.1 / 434.8 ± 57.2
**Ovarian cancer**		
Caov-3	ATCC/ IMDM	304.3 ± 24.5 / > 5000
COLO-704	DSMZ/ RPMI 1640	195.2 ± 28.1 / > 5000
EFO-21	DSMZ/ RPMI 1640	166.9 ± 20.6 / > 5000
EFO-27	DSMZ/ RPMI 1640	127.5 ± 16.2 / > 5000
FU-OV-1	DSMZ/DMEM Ham´s F12	> 5000 / > 5000
KURAMOCHI	JCRB/RPMI 1640	161.1 ± 26.8 / > 5000
MCAS	JCRB/MEM	575.9 ± 110.8 / > 5000
OAW-42	CLS/ DMEM	1451 ± 175 / > 5000
PA-1	JCRB/ MEM	615.2 ± 38.3 / 4173 ± 791
SKOV-3	ATCC/ McCoy´s 5a	176.4 ± 21.3 / > 5000
**Pancreas cancer**		
AsPC-1	CLS/ RPMI 1640	734.1 ± 80.9 / > 5000
Capan-1	CLS/RPMI 1640	311.2 ± 34.7 / > 5000
CAPAN-2	DSMZ/ RPMI 1640	143.7 ± 15.6 / > 5000
HUP-T3	DSMZ/ MEM	445.1 ± 39.5 / > 5000
HUP-T4	DSMZ/ MEM	361.6 ± 34.8 / > 5000
DAN-G	CLS/RPMI 1640	309.4 ± 48.2 / > 5000
Panc-1	CLS/ DMEM	1035 ± 139 / > 5000
PA-CLS 52	CLS/ MEM	335.1 ± 36.2 / > 5000
PA-TU-8902	DSMZ/ DMEM	235.4 ± 30.4 / > 5000

YAPC	DSMZ/ RPMI 1640	505.8 ± 62.7 / > 5000
**Prostate carcinoma**		
22RV1	DSMZ/ RPMI 1640 DMEM	560.1 ± 59.5 / > 5000
DU145	DSMZ/ RPMI 1640	666.1 ± 62.3 / > 5000
LNCAP	DSMZ/ RPMI 1640	395.6 ± 48.2 / > 5000
PC-3	DSMZ/ RPMI 1640 Ham´s F12	372.3 ± 38.1 / > 5000
VCaP	ATCC/ DMEM	464.3 ± 39.6 / > 5000
**Renal cell carcinoma**		
786-O	CLS/RPMI 1640	196.5 ± 22.6 / > 5000
769-P	CLS/RPMI 1640	290.1 ± 38.2 / 642.4 ± 75.9
A498	DSMZ/MEM	927.8 ± 112.9 / > 5000
A-704	CLS/ MEM	651.5 ± 129.2 / 1913 ± 285
ACHN	CLS/ MEM	525.7 ± 72.5 / 3698 ± 406
CAKI-1	DSMZ/McCoy´s 5a	489.1 ± 90.7 / > 5000
CAKI-2	DSMZ/ McCoy´s 5a	220.1 ± 14.4 / > 5000
CAL-54	DSMZ/DMEM + hydrocortisone + EGF	210.2 ± 15.1 / > 5000
CLS-439	CLS/ McCoy´s 5a	256.2 ± 35.4 / > 5000
UKF-RC-1	Cinatl/ IMDM	992.0 ± 109.3 / > 5000
**Retinoblastoma**		
RB247C	ECACC/ IMDM + insulin + 2-mercaptoethanol	715.5 ± 89.1 / > 5000
WERI-Rb1	ATCC/ RPMI 1640	> 5,000 / > 5000
Y79	ATCC/ RPMI 1640	1,824 ± 311 / > 5000
**Rhabdomyosarcoma**		
AX-OH-1	Koscielniak/ IMDM	425.6 ± 59.5 / 3619 ± 422
FL-OH-1	Koscielniak/ IMDM	> 5000 / > 5000
HA-OH-1	Koscielniak/ IMDM	56.5 ± 3.8 / 114.1 ± 8.6
HS-729	CLS/ DMEM	444.6 ± 85.3 / 2337 ± 281
KFR	Cinatl/ IMDM	870.3 ± 77.8 / > 5000
KYM-1	JCRB/DMEM Ham´s F12	2273 ± 393 / > 5000
RD	ATCC/ DMEM	719.0 ± 80.6 / > 5000
Rh28	Houghton/ IMDM	> 5000 / > 5000
Rh30	DSMZ/ RPMI 1640	483.4 ± 21.8 / > 5000
Rh36	Houghton/ IMDM	126.8 ± 13.2 / > 5000
Rh41	Houghton/ IMDM	358.5 ± 42.6 / > 5000
RMZ-RC2	University of Bologna/ DMEM	1501 ± 132 / > 5000
UKF-Rhb-1	Cinatl/ IMDM	224.0 ± 35.8 / 445.7 ± 50.3

**Thyroid cancer**		
8505C	DSMZ/ RPMI 1640	530.7 ± 58.3 / > 5000
BHT-101	DSMZ/DMEM	311.7 ± 44.6 / > 5000
B-CPAP	DSMZ/RPMI 1640	114.1 ± 10.0 / > 5000
MB-1	DSMZ/ IMDM	269.9 ± 37.4 / 583.5 ± 61.8
SW579	ATCC/ Leibovitz´s L15	178.5 ± 27.3 / 535.0 ± 65.9
SW-1736	CLS/ RPMI 1640	1361 ± 155 / > 5000
TCO-1	JCRB/DMEM	538.6 ± 92.4 / > 5000
**Urothelial carcinoma**		
5637	ATCC/ IMDM	126.0 ± 13.1 / 267.5 ± 33.8
647-V	DSMZ/DMEM	412.2 ± 58.5 / 712.9 ± 90.2
BFTC-905	DSMZ/ DMEM	268.2 ± 29.6 / > 5000
CAL 29	DSMZ/DMEM	665.1 ± 82.6 / > 5000
CLS-439	CLS/ RPMI 1640	271.5 ± 31.8 / > 5000
HB-CLS-1	CLS/ RPMI 1640	189.0 ± 24.4 / > 5000
HT-1376	ATCC/ IMDM	604.5 ± 71.8 / > 5000
JMSU-1	DSMZ/ RPMI 1640	425.1 ± 59.5 / > 5000
KU-19-19	DSMZ/RPMI 1640	179.5 ± 21.2 / 933.8 ± 110.4
RT112	ATCC/ IMDM	373.6 ± 39.9 / 2014 ± 228
RT4	ATCC/ IMDM	467.2 ± 59.3 / > 5000
T24	ATCC/ IMDM	> 5000 / > 5000
TCCSUP	ATCC/ MEM	831.9 ± 107.2 / > 5000
UM-UC-6	ECACC/ IMDM	47.4 ± 9.3 / 164.9 ± 45.0
VM-CUB1	DSMZ/ DMEM	271.3 ± 32.8 / 1936 ± 254
**Wilms' tumour (nephroblastoma)**		
SK-NEP-1	CLS/ McCoy´s 5a	347.4 ± 38.3 / 570.9 ± 70.5
WiT49	Bjerke/ DMEM F12	241.5 ± 34.8 / > 5000
WT-CLS1	CLS/ IMDM	718.1 ± 88.3 / > 5000

[1] Asterand (Hoyston, UK); ATCC (Manassas, VA, US); Bjerke, Dr. Lynn Bjerke (Institute for Cancer Research, Sutton, UK); Cinatl, Dr. Jindrich Cinatl (Institut für Medizinische Virologie, Frankfurt/M, Germany); CLS (CLS Cell Line Services, Eppelheim, Germany); DSMZ (Braunschweig, Germany), ECACC via Sigma-Aldrich Chemie GmbH (Munich, Germany); Edinger Institute, Institute of Neurology (Edinger Institute, Frankfurt /M, Germany), Eggert, Dr. Angelika Eggert (Universität Duisburg-Essen, Germany); Houghton, Dr. Peter J. Houghton (St. Jude's Children's Research Hospital, Memphis, TN); ICLC (Genova, Italy); JCRB, JCRB Cell Bank (Osaka, Japan); Koscielniak, Dr. Ewa Koscielniak (Olgahospital, Stuttgart, Germany); Naumann, Dr. Ulrike Naumann (Eberhard Karls-Universität; Tübingen, Germany); NIBSC, National Institute for Biological Standards and Control (South Mimms, UK); Pietsch, Dr. Torsten Pietsch (University of Bonn, Germany); von Laer, Dr. Dorothee von Laer (Medizinische Universität Innsbruck, Austria); Westermann, Dr. Frank

Westermann (DKFZ, Heidelberg, Germany); Wistar Institute (Philadelphia, , USA); Witt, Dr. Olaf Witt (DKFZ, Heidelberg, Germany)
2 DMEM, Dulbecco/Vogt Modified Eagle’s Minimal Essential Medium; DMEM Ham’s F12, 1:1 mixture of DMEM and Ham's F12 Nutrient Mixture; MEM, Eagle’s Minimal Essential Medium; RPMI 1640, Roswell Park Memorial Institute Medium 1640
The medium for all cell lines was supplemented with 10% FCS.

Suppl. Table S2. Sensitivity of parental neuroblastoma cells and their drug-adapted sub-lines to flubendazole as indicated by the concentrations that reduce cell viability by 50% (IC_{50}) or 90% (IC_{90}) indicated by MTT assay after a five day treatment period.

Cell line	IC_{50} (nM) flubendazole	IC_{90} (nM) flubendazole
**GI-ME-N**	**97.9 ± 14.7**	**640.8 ± 78.2**
GI-ME-N^{r}CDDP^{500}	206.9 ± 25.2 (2.11)[1]	614.6 ± 81.3 (0.96)[2]
GI-ME-N^{r}DOX^{5}	78.6 ± 8.9 (0.80)	265.1 ± 30.4 (0.41)
GI-ME-N^{r}VCR^{14}	471.8 ± 59.0 (4.82)	> 5000 (7.80)
**IMR-5**	**125.9 ± 7.0**	**266.3 ± 23.7**
IMR-5^{r}CARBO^{5000}	106.2 ± 2.7 (0.84)	188.3 ± 15.8 (0.71)
IMR-5^{r}CDDP^{1000}	144.4 ± 4.5 (1.36)	271.8 ± 36.0 (1.02)
IMR-5^{r}DACARB^{20}	179.9 ± 26.5 (1.43)	483.4 ± 81.7 (1.82)
IMR-5^{r}DOCE^{20}	143.2 ± 3.0 (1.14)	269.1 ± 27.9 (1.01)
IMR-5^{r}DOX^{20}	156.4 ± 15.6 (1.24)	1308 ± 157 (4.91)
IMR-5^{r}ETO^{100}	111.0 ± 5.2 (0.88)	188.3 ± 16.8 (0.71)
IMR-5^{r}GEMCI^{20}	82.6 ± 15.6 (0.67)	124.6 ± 15.1 (0.47)
IMR-5^{r}IRINO^{1000}	155.7 ± 19.2 (1.24)	375.2 ± 49.5 (1.41)
IMR-5^{r}MEL^{3000}	130.6 ± 4.3 (1.04)	395.4 ± 44.1 (1.48)
IMR-5^{r}OXALI^{4000}	89.6 ± 5.8 (0.71)	195.2 ± 22.1 (0.73)
IMR-5^{r}PCL^{20}	61.6 ± 4.7 (0.49)	119.4 ± 20.6 (0.45)
IMR-5^{r}TOPO^{20}	75.1 ± 16.7 (0.60)	138.3 ± 16.4 (0.52)
IMR-5^{r}VCR^{10}	314.7 ± 6.9 (2.50)	431.5 ± 49.6 (1.62)
IMR-5^{r}VINB^{20}	346.7 ± 20.2 (2.75)	> 5000 (> 18.78)
IMR-5^{r}VINOR^{20}	161.6 ± 4.8 (1.28)	261.5 ± 28.3 (0.98)
**IMR-32**	**97.2 ± 42.8**	**226.0 ± 23.1**
IMR-32^{r}BORTE^{10}	170.2 ± 20.8 (1.75)	267.1 ± 34.7 (1.18)
IMR-32^{r}CARBO^{1000}	337.1 ± 30.0 (3.47)	637.9 ± 45.4 (2.82)
IMR-32^{r}CDDP^{1000}	50.1 ± 2.7 (0.52)	97.9 ± 9.2 (0.43)
IMR-32^{r}DOX^{20}	313.2 ± 11.7 (3.22)	1582 ± 205 (7.00)
IMR-32^{r}ETO^{100}	237.3 ± 18.8 (2.44)	471.8 ± 29.5 (2.09)
IMR-32^{r}GEMCI^{25}	139.3 ± 4.0 (1.43)	260.6 ± 31.2 (1.15)
IMR-32^{r}MEL^{500}	126.6 ± 11.1 (1.30)	288.4 ± 36.6 (1.28)
IMR-32^{r}OXALI^{800}	216.1 ± 7.1 (2.22)	4187 ± 337 (18.53)
IMR-32^{r}TOPO^{8}	105.5 ± 15.7 (1.09)	246.3 ± 33.5 (1.09)
IMR-32^{r}VCR^{10}	317.8 ± 32.0 (3.27)	951.7 ± 82.6 (4.21)
IMR-32^{r}VINOR^{5}	254.0 ± 12.1 (2.61)	618.2 ± 42.5 (2.74)
**NGP**	**303.7 ± 45.3**	**1781 ± 451**
NGP^{r}BORTE^{20}	238.6 ± 36.1 (0.79)	527.2 ± 84.9 (0.30)
NGP^{r}CARBO^{2000}	175.1 ± 29.2 (0.58)	499.3 ± 62.7 (0.28)
NGP^{r}CDDP^{1000}	299.0 ± 33.6 (0.98)	629.2 ± 89.4 (0.35)
NGP^{r}DACARB^{18}	270.4 ± 67.8 (0.89)	1873 ± 382 (1.05)
NGP^{r}DOCE^{20}	468.4 ± 59.2 (1.54)	1672 ± 354 (0.94)
NGP^{r}DOX^{20}	201.9 ± 37.6 (0.66)	> 5000 (2.81)
NGP^{r}ETO^{200}	146.2 ± 17.8 (0.48)	372.5 ± 46.4 (0.21)
NGP^{r}GEMCI^{20}	186.7 ± 31.4 (0.61)	1227 ± 199 (0.69)
NGP^{r}IRINO^{1000}	177.6 ± 28.3 (0.58)	451.8 ± 70.3 (0.25)

NGP^{r}MEL^{2000}	325.2 ± 40.8 (1.07)	1237 ± 196 (0.69)
NGP^{r}OXALI^{4000}	208.3 ± 31.4 (0.69)	531.7 ± 99.6 (0.30)
NGP^{r}PCL^{20}	411.3 ± 69.0 (1.35)	1065 ± 174 (0.60)
NGP^{r}TOPO^{20}	269.2 ± 47.4 (0.89)	1773 ± 308 (1.00)
NGP^{r}VCR^{100}	513.0 ± 62.1 (1.69)	> 5000 (> 2.81)
NGP^{r}VINB^{20}	362.5 ± 89.4 (1.19)	> 5000 (> 2.81)
NGP^{r}VINOR^{20}	700.8 ± 113.6 (2.31)	> 5000 (> 2.81)
**NLF**	**141.0 ± 15.6**	**261.9 ± 77.0**
NLF^{r}CARBO^{5000}	76.4 ± 9.1 (0.54)	203.2 ± 15.3 (0.78)
NLF^{r}CDDP^{1000}	94.8 ± 12.1 (0.67)	305.1 ± 38.2 (1.16)
NLF^{r}DACARB^{20}	102.8 ± 10.6 (0.73)	151.6 ± 19.2 (0.58)
NLF^{r}DOCE^{20}	153.0 ± 16.1 (1.09)	180.5 ± 22.7 (0.69)
NLF^{r}DOX^{40}	73.9 ± 11.6 (0.52)	163.6 ± 14.6 (0.62)
NLF^{r}ETO^{100}	164.9 ± 20.6 (1.17)	280.9 ± 34.7 (1.07)
NLF^{r}GEMCI^{20}	58.2 ± 4.9 (0.41)	75.7 ± 9.8 (0.29)
NLF^{r}IRINO^{1000}	87.3 ± 12.6 (0.62)	180.2 ± 19.4 (0.69)
NLF^{r}MEL^{3000}	112.2 ± 15.1 (0.80)	271.6 ± 30.3 (1.04)
NLF^{r}OXALI^{4000}	131.5 ± 17.0 (0.93)	376.3 ± 51.6 (1.44)
NLF^{r}PCL^{20}	51.8 ± 6.2 (0.37)	157.4 ± 13.8 (0.60)
NLF^{r}TOPO^{10}	132.3 ± 15.8 (0.94)	247.1 ± 28.9 (0.94)
NLF^{r}VCR^{10}	183.5 ± 32.1 (1.30)	398.1 ± 50.0 (1.52)
NLF^{r}VINB^{10}	444.8 ± 32.7 (3.15)	723.3 ± 122.4 (2.76)
NLF^{r}VINOR^{10}	208.6 ± 20.2 (1.48)	345.1 ± 29.3 (1.32)
**SHEP**	**225.8 ± 8.3**	**426.1 ± 12.9**
SHEP^{r}CDDP^{1000}	176.9 ± 20.6 (0.78)	595.3 ± 71.0 (1.40)
SHEP^{r}DOX^{100}	209.1 ± 23.7 (0.93)	516.4 ± 62.8 (1.21)
SHEP^{r}ETO^{100}	162.0 ± 18.5 (0.72)	559.7 ± 73.1 (1.31)
SHEP^{r}VCR^{20}	302.1 ± 34.8 (1.34)	573.7 ± 67.2 (1.35)
**SH-SY5Y**	**166.8 ± 14.6**	**479.7 ± 58.6**
SH-SY5Y^{r}CDDP^{200}	100.7 ± 13.1 (0.60)	331.6 ± 38.7 (0.69)
SH-SY5Y^{r}DOX^{20}	98.7 ± 11.3 (0.59)	642.7 ± 75.1 (1.34)
SH-SY5Y^{r}VCR^{10}	227.6 ± 41.3 (1.36)	582.8 ± 86.2 (1.21)
**SK-N-AS**	**159.3 ± 38.8**	**352.3 ± 84.0**
SK-N-AS^{r}CARBO^{2000}	55.4 ± 10.1 (0.35)	189.6 ± 23.8 (0.54)
SK-N-AS^{r}CDDP^{1000}	70.7 ± 13.4 (0.44)	252.1 ± 65.6 (0.72)
SK-N-AS^{r}DACARB^{16}	74.2 ± 10.6 (0.47)	150.5 ± 23.1 (0.43)
SK-N-AS^{r}DOCE^{10}	187.5 ± 26.4 (1.18)	> 5000 (> 14.19)
SK-N-AS^{r}DOX^{10}	36.2 ± 4.6 (0.23)	72.8 ± 10.1 (0.21)
SK-N-AS^{r}ETO^{100}	75.5 ± 17.2 (0.47)	1025 ± 174 (2.91)
SK-N-AS^{r}GEMCI^{10}	86.8 ± 12.6 (0.54)	189.2 ± 23.9 (0.54)
SK-N-AS^{r}IRINO^{500}	165.2 ± 23.6 (1.04)	276.1 ± 34.1 (0.78)
SK-N-AS^{r}MEL^{1000}	122.4 ± 19.6 (0.77)	290.3 ± 41.7 (0.82)
SK-N-AS^{r}OXALI^{4000}	342.7 ± 49.3 (2.15)	> 5000 (> 14.19)
SK-N-AS^{r}PCL^{20}	105.7 ± 20.1 (0.66)	639.4 ± 89.0 (1.81)
SK-N-AS^{r}TOPO^{20}	192.1 ± 24.8 (1.21)	297.9 ± 35.2 (0.85)
SK-N-AS^{r}VCR^{20}	126.4 ± 18.1 (0.79)	279.2 ± 33.5 (0.79)
SK-N-AS^{r}VINB^{10}	115.0 ± 17.8 (0.72)	191.3 ± 25.7 (0.54)
SK-N-AS^{r}VINOR^{20}	273.6 ± 33.8 (1.72)	676.2 ± 75.7 (1.92)
**UKF-NB-1**	**243.6 ± 29.3**	**567.1 ± 72.8**

UKF-NB-1^{r}CDDP^{1000}	271.4 ± 29.8 (1.11)	479.7 ± 51.4 (0.85)
UKF-NB-1^{r}VCR^{10}	303.7 ± 47.6 (1.25)	891.4 ± 163.9 (1.57)
**UKF-NB-2**	**128.0 ± 22.3**	**283.4 ± 35.1**
UKF-NB-2^{r}CARBO^{2000}	144.3 ± 16.7 (1.13)	486.1 ± 89.6 (1.72)
UKF-NB-2^{r}CDDP^{1000}	115.5 ± 14.3 (0.90)	392.6 ± 46.9 (1.39)
UKF-NB-2^{r}DACARB^{4}	103.4 ± 19.9 (0.81)	307.3 ± 46.1 (1.08)
UKF-NB-2^{r}DOCE^{10}	166.5 ± 39.8 (1.30)	489.2 ± 65.5 (1.73)
UKF-NB-2^{r}DOX^{20}	422.0 ± 27.7 (3.30)	660.3 ± 81.5 (2.33)
UKF-NB-2^{r}ETO^{40}	56.7 ± 9.6 (0.44)	189.2 ± 13.3 (0.67)
UKF-NB-2^{r}IRINO^{400}	272.5 ± 36.2 (2.13)	> 5000 (> 17.64)
UKF-NB-2^{r}MEL^{600}	214.2 ± 17.1 (1.67)	568.4 ± 60.7 (2.01)
UKF-NB-2^{r}Nutlin^{10µM}	191.9 ± 21.8 (1.50)	562.7 ± 71.5 (1.99)
UKF-NB-2^{r}OXALI^{600}	125.6 ± 12.3 (0.98)	291.7 ± 36.7 (1.03)
UKF-NB-2^{r}PCL^{20}	138.4 ± 5.2 (1.08)	213.6 ± 18.4 (0.75)
UKF-NB-2^{r}TOPO^{10}	161.3 ± 14.5 (1.26)	641.7 ± 72.8 (2.26)
UKF-NB-2^{r}VCR^{10}	162.4 ± 15.8 (1.27)	491.8 ± 61.3 (1.74)
UKF-NB-2^{r}VINB^{10}	132.6 ± 18.3 (1.04)	272.4 ± 20.9 (0.96)
UKF-NB-2^{r}VINOR^{10}	178.7 ± 13.8 (1.40)	299.8 ± 48.2 (1.06)
**UKF-NB-3**	**48.0 ± 7.9**	**102.3 ± 12.7**
UKF-NB-3^{r}BORTE^{20}	70.7 ± 14.6 (1.47)	301.2 ± 55.1 (2.94)
UKF-NB-3^{r}CABAZI^{2}	140.2 ± 18.1 (2.92)	> 5000 (> 48.88)
UKF-NB-3^{r}CARBO^{2000}	106.4 ± 2.0 (2.22)	215.8 ± 21.9 (2.11)
UKF-NB-3^{r}CDDP^{1000}	63.6 ± 21.7 (1.33)	122.1 ± 16.7 (1.19)
UKF-NB-3^{r}DACARB^{8}	100.5 ± 12.8 (2.09)	147.3 ± 18.6 (1.44)
UKF-NB-3^{r}DOCE^{20}	69.3 ± 15.4 (1.44)	125.3 ± 17.1 (1.22)
UKF-NB-3^{r}DOX^{20}	179.8 ± 8.6 (3.75)	241.6 ± 31.4 (2.36)
UKF-NB-3^{r}ETO^{200}	252.5 ± 33.9 (5.26)	538.2 ± 66.1 (5.26)
UKF-NB-3^{r}GEMCI^{10}	57.7 ± 3.4 (1.20)	227.4 ± 49.8 (2.22)
UKF-NB-3^{r}IRINO^{800}	160.0 ± 21.1 (3.33)	187.7 ± 25.3 (1.83)
UKF-NB-3^{r}ME^{1000}	575.8 ± 69.1 (12.00)	2072 ± 229 (20.25)
UKF-NB-3^{r}MEL^{2000}	196.0 ± 14.7 (4.08)	816.7 ± 98.2 (7.98)
UKF-NB-3^{r}Nutlin^{10µM}	201.6 ± 26.0 (4.20)	511.3 ± 120.4 (5.00)
UKF-NB-3^{r}NVP-TAE684^{500}	281.7 ± 29.4 (5.89)	559.0 ± 61.7 (5.46)
UKF-NB-3^{r}OXALI^{2000}	143.8 ± 17.8 (3.00)	541.5 ± 42.6 (5.29)
UKF-NB-3^{r}PCL^{20}	93.4 ± 8.0 (1.95)	189.6 ± 21.8 (1.85)
UKF-NB-3^{r}RITA^{10µM}	135.2 ± 18.9 (2.82)	244.3 ± 28.5 (2.39)
UKF-NB-3^{r}TOPO^{15}	155.8 ± 3.9 (3.25)	264.1 ± 17.5 (2.58)
UKF-NB-3^{r}VCR^{10}	214.1 ± 21.4 (4.46)	552.3 ± 60.9 (5.40)
UKF-NB-3^{r}VINB^{10}	163.3 ± 20.8 (3.40)	456.2 ± 50.7 (4.46)
UKF-NB-3^{r}VINOR^{20}	364.5 ± 28.6 (7.59)	1325 ± 144 (12.95)
UKF-NB-3^{r}YM155^{50nM}	81.3 ± 11.9 (1.69)	202.6 ± 25.3 (1.98)
**UKF-NB-4**	**134.9 ± 33.4**	**276.8 ± 22.0**
UKF-NB-4^{r}CDDP^{1000}	108.4 ± 16.5 (0.80)	351.8 ± 30.3 (1.27)
UKF-NB-4^{r}DOX^{350}	215.7 ± 76.1 (1.60)	841.7 ± 285.7 (3.04)
UKF-NB-4^{r}VCR^{50}	242.3 ± 4.8 (1.80)	496.0 ± 63.7 (1.79)
**UKF-NB-6**	**207.3 ± 19.3**	**335.6 ± 49.9**
UKF-NB-6^{r}CARBO^{2000}	106.0 ± 6.7 (0.51)	329.8 ± 35.5 (0.98)
UKF-NB-6^{r}CDDP^{1000}	219.2 ± 10.0 (1.06)	557.1 ± 43.8 (1.66)
UKF-NB-6^{r}DACARB^{8}	165.9 ± 18.6 (0.80)	291.6 ± 30.5 (0.87)

UKF-NB-6^{r}DOCE^{10}	195.7 ± 6.9 (0.94)	263.7 ± 18.6 (0.79)
UKF-NB-6^{r}DOX^{20}	214.2 ± 19.4 (1.03)	404.1 ± 49.4 (1.20)
UKF-NB-6^{r}ETO^{200}	233.2 ± 30.0 (1.12)	379.6 ± 26.8 (1.13)
UKF-NB-6^{r}GEMCI^{10}	261.1 ± 25.2 (1.26)	577.2 ± 36.4 (1.72)
UKF-NB-6^{r}IRINO^{1000}	236.7 ± 26.2 (1.14)	340.1 ± 24.7 (1.01)
UKF-NB-6^{r}MEL^{2000}	275.5 ± 17.1 (1.33)	513.5 ± 38.3 (1.53)
UKF-NB-6^{r}OXALI^{4000}	139.7 ± 15.4 (0.67)	242.9 ± 17.2 (0.72)
UKF-NB-6^{r}PCL^{20}	105.8 ± 17.9 (0.51)	271.8 ± 22.5 (0.81)
UKF-NB-6^{r}TOPO^{20}	177.5 ± 18.2 (0.86)	581.6 ± 61.2 (1.73)
UKF-NB-6^{r}VCR^{10}	435.3 ± 63.8 (2.10)	991.7 ± 114.6 (2.96)
UKF-NB-6^{r}VINB^{10}	341.1 ± 19.5 (1.65)	545.8 ± 30.3 (1.63)
UKF-NB-6^{r}VINOR^{40}	167.2 ± 10.4 (0.81)	288.9 ± 19.0 (0.86)
UKF-NB-6^{r}YM155^{20nM}	176.9 ± 23.5 (0.85)	382.6 ± 56.6 (1.14)

[1] IC_{50} resistant sub-line/ IC_{50} parental cell line
[2] IC_{90} resistant sub-line/ IC_{90} parental cell line

Suppl. Table S3. Flubendazole concentrations that reduce viability by 50% (IC_{50}) or 90% (IC_{90}) in primary neuroblastoma cells as determined by MTT assay after a five day treatment period.

	IC_{50} (nM) flubendazole	IC_{90} (nM) flubendazole
isolate 1	54.7 ± 12.7	612.2 ± 98.9
isolate 2	235.2 ± 30.0	522.8 ± 78.4
isolate 3	190.2 ± 14.7	573.5 ± 49.8
isolate 4	117.6 ± 19.3	599.8 ± 44.8
isolate 5	135.1 ± 12.6	464.7 ± 67.2

Suppl. Table S4. Anti-cancer effects of flubendazole in ABCB1- and ABCG2-expressing cells. Concentrations that reduce cell viability by 50% (IC_{50}) have been determined by MTT assay after a five day treatment period.

Cell line	IC_{50} (µM) flubendazole	IC_{50} (ng/ml) vincristine[1]	IC_{50} (ng/ml) mitoxantrone[2]
UKF-NB-3	46.1 ± 6.4	0.204 ± 0.022	
+ verapamil (92 ± 10%)[3]	40.1 ± 6.7	0.151 ± 0.013	
...^{r}VCR[10] [4]	201.2 ± 17.5	61.1 ± 10.8	
+ verapamil (89 ± 11%)	194.0 ± 9.3	1.300 ± 0.211	
...^{ABCB1} [5]	63.2 ± 8.5	21.5 ± 3.0	
+ verapamil (93 ± 6%)	52.9 ± 6.2	0.143 ± 0.012	
...^{Cer2} [6]	55.6 ± 7.1	0.292 ± 0.033	
+ verapamil (90 ± 8%)	48.2 ± 7.9	0.276 ± 0.030	
UKF-NB-3	45.0 ± 5.3		0.118 ± 0.014
+ WK-X-34 (99 ± 9%)[7]	46.3 ± 7.9		0.100 ± 0.09
...^{ABCG2} [8]	68.5 ± 12.4		45.2 ± 7.5
+ WK-X-34 (105 ± 7%)	53.2 ± 12.2		0.160 ± 0.016
...^{iG2} [9]	44.8 ± 4.9		0.143 ± 0.017
+ WK-X-34 (102 ± 5%)	37.5 ± 3.5		0.138 ± 0.017

[1] cytotoxic ABCB1 substrate; [2] cytotoxic ABCG2 substrate; [3] verapamil (10µM), ABCB1 inhibitor (cell viability in the presence of verapamil alone); [4] cell line characterised by high ABCB1 expression; [5] UKF-NB-3 cells transduced with a lentiviral vector encoding for ABCB1; [6] UKF-NB-3 cells transduced with a vector control corresponding to UKF-NB-3^{ABCB1}; [7] WK-X-34 (1µM) ABCG2 inhibitor (cell viability in the presence of WK-X-34 alone); [8] UKF-NB-3 cells transduced with a lentiviral vector encoding for BCRP/ABCG2; [9] UKF-NB-3 cells transduced with a vector control corresponding to UKF-NB-3^{ABCG2}

Suppl. Table S5. Effects of wild-type p53 depletion on the flubendazole sensitivity of neuroblastoma cells. p53 was depleted using a lentiviral vector encoding for p53 shRNA. Concentrations that reduce cell viability by 50% (IC_{50}) or 90% (IC_{90}) were determined by MTT assay after a five day incubation period and ratios of the flubendazole IC_{50}s and IC_{90}s in the p53-depleted cells vs. the parental cells were calculated.

Cell line	IC_{50} (nM)/IC_{90} (nM) flubendazole	IC_{50} transduced sub-line/ IC_{50} parental cell line	IC_{90} transduced sub-line/ IC_{90} parental cell line
IMR-32	97.2 ± 42.8 / 186.0 ± 23.1		
...^{p53shRNA}[1]	188.5 ± 13.7 / 298.2 ± 30.2	1.9	1.6
...^{scrl}[2]	71.1 ± 15.6 / 157.2 ± 20.5	0.7	0.8
LAN-5	249.0 ± 33.7 / 674.8 ± 75.5		
...^{p53shRNA}[1]	556.6 ± 87.2 / > 5000	2.2	> 7.4
...^{scr}[2]	235.8 ± 18.1 / 723.6 ± 69.6	0.9	1.1
NB-S-124	75.2 ± 20.2 / 159.5 ± 8.4		
...^{p53shRNA}[1]	515.1 ± 104.0 / 4889 ± 225	6.8	30.7
...^{scr}[2]	74.4 ± 8.4 / 140.6 ± 10.1	1.0	0.9
SH-SY5Y	166.8 ± 14.6 / 479.7 ± 58.6		
...^{p53shRNA}[1]	323.0 ± 28.2 / 2501 ± 396	1.9	5.2
...^{scr}[2]	205.1 ± 44.3 / 489.8 ± 70.5	1.2	1.0
UKF-NB-2	128.0 ± 22.3 / 283.4 ± 35.1		
...^{p53shRNA}[1]	247.1 ± 26.4 / 837.9 ± 101.2	1.9	3.0
...^{scr}[2]	141.1 ± 12.6 / 362.5 ± 69.2	1.1	1.3
UKF-NB-3	48.0 ± 7.9 / 85.8 ± 6.5		
...^{p53shRNA}[1]	174.2 ± 22.7 / 4338 ± 527	3.6	50.6
...^{scr}[2]	56.3 ± 4.1 / 95.6 ± 10.2	1.2	1.1

[1] p53-depleted, i.e. transduced with a lentiviral vector expressing p53shRNA
[2] vector control, i.e. transduced with the vector expressing scrambled, non-targeting shRNA

Suppl. Table S6. Effects of flubendazole and the MDM2 antagonist nutlin-3 on neuroblastoma cell viability (% non-treated control).

		nutlin-3 (1.25µM)		nutlin-3 (0.625µM)	
	flubendazole		+ flubendazole		+flubendazole
flubendazole 80nM					
UKF-NB-3	21.91 ± 1.42	60.51 ± 16.07	0	72.60 ± 3.53	0
UKF-NB-3^{r}VCR^{10}	93.04 ± 2.23	78.23 ± 10.10	88.84 ± 3.96	77.56 ± 16.45	94.75 ± 13.80
UKF-NB-3^{scr} [1]	18.81 ± 2.06	51.97 ± 15.50	0	71.03 ± 6.22	0
UKF-NB-3^{p53shRNA} [2]	97.99 ± 3.62	81.54 ± 8.61	66.22 ± 2.57	92.00 ± 10.09	78.82 ± 11.35
flubendazole 40nM					
UKF-NB-3	60.80 ± 13.48	60.51 ± 16.07	6.76 ± 2.23	72.60 ± 3.53	29.96 ± 6.26
UKF-NB-3^{r}VCR^{10}	88.46 ± 9.93	78.23 ± 10.10	89.02 ± 17.54	77.56 ± 16.45	87.52 ± 2.01
UKF-NB-3^{scr}	73.93 ± 11.65	51.97 ± 15.50	17.63 ± 0.77	71.03 ± 6.22	35.37 ± 11.44
UKF-NB-3^{p53shRNA}	104.55 ± 3.02	81.54 ± 8.61	78.93 ± 2.60	92.00 ± 10.09	90.22 ± 3.69

[1] UKF-NB-3 cells transduced with a control vector [2] UKF-NB-3 transduced with a lentiviral vector expressing p53 shRNA

Suppl. Table S7. Anti-cancer effects of flubendazole dissolved in DMSO or as (2-hydroxypropyl)-β-cyclodextrin (CD) preparation. Concentrations that reduce cell viability by 50% (IC_{50}) were determined by MTT assay after a five day treatment period.

	IC_{50} (nM) flubendazole		
		(2-hydroxypropyl)-β-cyclodextrin complex	
Cell line	DMSO	freshly prepared	after 8 weeks
UKF-NB-3	48.0 ± 7.9	56.29 ± 11.37	53.33 ± 9.07

Suppl. Table S8. Flubendazole or mebendazole concentrations that inhibit viability of the investigated cell lines by 50% (IC_{50}) as indicated by MTT assay after a five day incubation period.

	IC_{50} (nM)	
Cell line	flubendazole	mebendazole
Colorectal carcinoma		
HT29	272.7 ± 10.1	433.3 ± 10.3
LoVo	352.6 ± 21.0	304.0 ± 25.3
RKO	252.1 ± 9.4	191.6 ± 6.0
Ewing's sarcoma		
A673	399.5 ± 15.3	323.5 ± 20.4
RD-ES	473.9 ± 18.0	417.6 ± 17.4
Glioma		
G62	355.3 ± 13.6	289.8 ± 14.6
GMS-10	582.3 ± 20.7	565.8 ± 25.1
LN229	363.0 ± 31.9	432.4 ± 38.1
U138MG	> 5,000	> 5,000
Hepatoma		
HepG2	362.7 ± 19.3	343.1 ± 8.8
Leukaemia/lymphoma		
JURKAT	123.3 ± 10.5	112.2 ± 10.6
L-428	178.9 ± 23.2	128.6 ± 21.9

Lung carcinoma		
HCC-44	208.8 ± 20.4	149.7 ± 7.1
HCC-827	225.1 ± 25.6	176.0 ± 16.2
Medulloblastoma		
D283Med	121.6 ± 9.3	104.0 ± 12.9
Daoy	176.6 ± 13.1	160.1 ± 24.8
MHH-Med-1	445.0 ± 32.2	452.7 ± 15.1
Melanoma		
Colo-679	477.2 ± 33.4	447.6 ± 36.5
IGR-39	317.4 ± 14.2	270.6 ± 20.8
MelHO	422.3 ± 30.1	358.8 ± 11.9
MelJuso	1849.4 ± 278.6	> 5,000
MeWo	381.5 ± 39.2	340.0 ± 32.1
RVH-421	614.1 ± 48.6	604.2 ± 45.6
SK-Mel-30	490.8 ± 20.6	403.6 ± 19.3
Neuroblastoma		
IMR-32	97.2 ± 42.8	122.8 ± 12.8
SH-SY5Y	166.8 ± 14.6	152.5 ± 14.5
UKF-NB-2	128.0 ± 22.3	142.7 ± 16.6
UKF-NB-3	48.0 ± 7.9	40.8 ± 6.1
UKF-NB-6	207.3 ± 19.3	224.0 ± 22.6
Prostate carcinoma		
22RV1	560.1 ± 59.5	395.9 ± 46.1
DU145	666.1 ± 62.3	562.7 ± 81.8

LNCaP	395.6 ± 48.19	293.7 ± 62.0
PC-3	372.3 ± 38.1	332.5 ± 33.3
Rhabdomyosarcoma		
KFR	870.3 ± 77.8	1149.3 ± 58.8
Rh30	483.4 ± 21.8	361.4 ± 24.0
Thyroid carcinoma		
8505C	530.7 ± 58.3	377.5 ± 49.4
Urothelial carcinoma		
HT-1376	604.5 ± 71.8	600.9 ± 28.4
RT112	373.6 ± 39.9	345.1 ± 35.8

Suppl. Table S9. Sensitivity of parental neuroblastoma cells and their drug-adapted sub-lines to flubendazole or mebendazole as indicated by the concentration that reduces cell viability by 50% (IC_{50}) indicated by MTT assay after a five day treatment period and ratios flubendazole or mebendazole IC_{50}s drug-adapted sub-lines/parental cell lines.

Cell line	IC_{50} (nM) flubendazole	IC_{50} flubendazole drug-adapted sub-line/ parental cell line	IC_{50} (nM) mebendazole	IC_{50} mebendazole drug-adapted sub-line/ parental cell line
IMR-32	97.2 ± 42.8		122.8 ± 12.8	
...^{r}DOX^{20}	313.2 ± 11.7	3.2	272.8 ± 33.2	2.2
...^{r}ETO^{100}	237.3 ± 18.8	2.4	165.7 ± 13.2	1.3
...^{r}MEL^{500}	126.6 ± 11.1	1.3	120.8 ± 12.2	1.0
...^{r}TOPO^{8}	105.5 ± 15.7	1.1	92.7 ± 18.4	0.8
...^{r}VCR^{10}	317.8 ± 32.0	3.3	278.1 ± 14.5	2.3
UKF-NB-2	128.0 ± 22.3		142.7 ± 16.6	
...^{r}CDDP^{1000}	115.5 ± 14.3	0.9	92.3 ± 8.6	0.6
...^{r}DOX^{20}	422.0 ± 27.7	3.3	383.7 ± 30.0	2.7
...^{r}ETO^{40}	56.7 ± 9.6	0.4	119.4 ± 13.9	0.8
...^{r}MEL^{600}	214.2 ± 17.1	1.7	181.5 ± 18.5	1.3
...^{r}TOPO^{10}	161.3 ± 14.5	1.3	158.6 ± 15.3	1.1
...^{r}VCR^{10}	162.4 ± 15.8	1.3	176.8 ± 20.4	1.2
UKF-NB-3	48.0 ± 7.9		40.8 ± 6.1	
...^{r}CDDP^{1000}	78.9 ± 5.6	1.6	60.7 ± 5.4	1.5
...^{r}ETO^{200}	252.5 ± 33.9	5.3	169.9 ± 16.4	4.2
...^{r}MEL^{2000}	196.0 ± 14.7	4.1	152.1 ± 13.0	3.7
...^{r}PCL^{20}	93.4 ± 8.0	1.9	67.1 ± 11.6	1.6

...^{r}VCR^{10}	214.1 ± 21.4	4.5	168.8 ± 9.1	4.1
...^{r}VINB^{10}	163.3 ± 20.8	3.4	166.4 ± 15.9	4.1
...^{r}VINOR^{20}	364.5 ± 28.6	7.6	304.2 ± 25.9	7.5
UKF-NB-6	207.3 ± 19.3		224.0 ± 22.6	
...^{r}CDDP^{1000}	219.2 ± 10.0	1.1	245.0 ± 25.8	1.1
...^{r}DOX^{20}	214.2 ± 19.4	1.0	211.7 ± 23.1	0.9
...^{r}ETO^{200}	233.2 ± 30.0	1.1	255.9 ± 30.1	1.1
...^{r}MEL^{2000}	275.5 ± 17.1	1.3	253.0 ± 22.9	1.1
...^{r}PCL^{20}	105.8 ± 17.9	0.5	166.2 ± 15.9	0.7
...^{r}TOPO^{20}	177.5 ± 18.2	0.9	180.8 ± 17.8	0.8
...^{r}VCR^{10}	435.3 ± 63.8	2.1	415.3 ± 47.0	1.9

Suppl. Table S10. Drug concentrations that inhibit the viability of parental cancer cell lines and their drug-adapted sub-lines by 50% (IC$_{50}$) as indicated by MTT assay after 5 day incubation.

Cell line	IC$_{50}$ cisplatin
GI-ME-N	47.1 ± 8.4 ng/mL
GI-ME-N^{r}CDDP^{500}	1384 ± 181 ng/mL
Cell line	IC$_{50}$ doxorubicin
GI-ME-N	0.81 ± 0.19 ng/mL
GI-ME-N^{r}DOX^{5}	10.11 ± 2.2 ng/mL
Cell line	IC$_{50}$ vincristine
GI-ME-N	0.06 ± 0.01 ng/mL
GI-ME-N^{r}VCR^{14}	42.19 ± 9.03 ng/mL
Cell line	IC$_{50}$ carboplatin
IMR-5	914.63 ± 45.92 ng/mL
IMR-5^{r}CARBO^{5000}	5484 ± 424 ng/mL
Cell line	IC$_{50}$ cisplatin
IMR-5	158.00 ± 5.25 ng/mL
IMR-5^{r}CDDP^{1000}	1214 ± 170 ng/mL
Cell line	IC$_{50}$ dacarbazine
IMR-5	17.83 µg/mL
IMR-5^{r}DACARB^{20}	73.67 µg/mL
Cell line	IC$_{50}$ docetaxel
IMR-5	0.70 ± 0.02 ng/mL
IMR-5^{r}DOCE^{20}	59.41 ± 4.18 ng/mL
Cell line	IC$_{50}$ doxorubicin
IMR-5	6.76 ± 1.53 ng/mL
IMR-5^{r}DOX^{20}	365.18 ± 50.97 ng/mL
Cell line	IC$_{50}$ etoposide
IMR-5	22.13 ± 1.16 ng/mL
IMR-5^{r}ETO^{100}	278.63 ± 19.75 ng/mL
Cell line	IC$_{50}$ gemcitabine
IMR-5	3.34 ± 0.41 ng/mL
IMR-5^{r}GEMCI^{20}	53.31 ± 6.31 ng/mL
Cell line	IC$_{50}$ irinotecan
IMR-5	342.78 ± 61.27 ng/mL
IMR-5^{r}IRINO^{1000}	39,224 ± 5,618 ng/mL
Cell line	IC$_{50}$ melphalan

IMR-5	915.80 ± 66.63 ng/mL
IMR-5^{r}MEL^{3000}	8453.53 ± 184.97 ng/mL
Cell line	IC_{50} oxaliplatin
IMR-5	552.25 ± 23.15 ng/mL
IMR-5^{r}OXALI^{4000}	5495 ± 143 ng/mL
Cell line	IC_{50} paclitaxel
IMR-5	0.77 ± 0.04 ng/mL
IMR-5^{r}PCL^{20}	42.61 ± 14.94 ng/mL
Cell line	IC_{50} topotecan
IMR-5	0.83 ± 0.15 ng/mL
IMR-5^{r}TOPO^{20}	98.50 ± 20.27 ng/mL
Cell line	IC_{50} vincristine
IMR-5	0.46 ± 0.04 ng/mL
IMR-5^{r}VCR^{10}	31.41 ± 2.69 ng/mL
Cell line	IC_{50} vinblastine
IMR-5	1.32 ± 0.26 ng/mL
IMR-5^{r}VINB^{20}	39.40 ± 6.67 ng/mL
Cell line	IC_{50} vinorelbine
IMR-5	3.48 ± 0.52 ng/mL
IMR-5^{r}VINOR^{20}	25.54 ± 4.16 ng/mL
Cell line	IC_{50} bortezomib
IMR-32	0.78 ± 0.20 nM
IMR-32^{r}BORTE^{10}	18.16 ± 2.05 nM
Cell line	IC_{50} carboplatin
IMR-32	641.87 ± 144.44 ng/mL
IMR-32^{r}CARBO^{4000}	11158 ± 3617 ng/mL
Cell line	IC_{50} cisplatin
IMR-32	190.40 ± 27.66 ng/mL
IMR-32^{r}CDDP^{1000}	3998 ± 106 ng/mL
Cell line	IC_{50} doxorubicin
IMR-32	4.18 ± 2.14 ng/mL
IMR-32^{r}DOX^{20}	58.15 ± 3.54 ng/mL
Cell line	IC_{50} etoposide
IMR-32	39.18 ± 5.93 ng/mL
IMR-32^{r}ETO^{100}	245 ± 28 ng/mL
Cell line	IC_{50} gemcitabine
IMR-32	2.48 ± 0.37 ng/mL

IMR-32^{r}GEMCI^{25}	67.03 ± 12.51 ng/mL
Cell line	IC$_{50}$ melphalan
IMR-32	242 ± 45 ng/mL
IMR-32^{r}MEL^{500}	1169 ± 125 ng/mL
Cell line	IC$_{50}$ oxaliplatin
IMR-32	944.20 ± 45.61 ng/mL
IMR-32^{r}OXALI^{4000}	5673 ± 530 ng/mL
Cell line	IC$_{50}$ topotecan
IMR-32	1.77 ± 0.27 ng/mL
IMR-32^{r}TOPO^{8}	22.79 ± 5.55 ng/mL
Cell line	IC$_{50}$ vincristine
IMR-32	0.28 ± 0.07 ng/mL
IMR-32^{r}VCR^{10}	29.55 ± 3.79 ng/mL
Cell line	IC$_{50}$ vinorelbine
IMR-32	0.37 ± 0.02 ng/mL
IMR-32^{r}VINOR^{5}	6.94 ± 0.70 ng/mL
Cell line	IC$_{50}$ bortezomib
NGP	3.83 ± 0.49 nM
NGP^{r}BORTE^{20}	28.83 ± 6.94 nM
Cell line	IC$_{50}$ carboplatin
NGP	716 ± 105 ng/mL
NGP^{r}CARBO^{2000}	2402 ± 366 ng/mL
Cell line	IC$_{50}$ cisplatin
NGP	179.2 ± 26.6 ng/mL
NGP^{r}CDDP^{1000}	2016 ± 327 ng/mL
Cell line	IC$_{50}$ dacarbazine
NGP	2.83 ± 0.43 µg/mL
NGP^{r}DACARB^{18}	55.84 ± 10.46 µg/mL
Cell line	IC$_{50}$ docetaxel
NGP	0.011 ± 0.006 ng/mL
NGP^{r}DOCE^{20}	103.2 ± 15.8 ng/mL
Cell line	IC$_{50}$ doxorubicin
NGP	7.56 ± 1.07 ng/mL
NGP^{r}DOX^{20}	160.2 ± 24.8 ng/mL
Cell line	IC$_{50}$ etoposide
NGP	48.31 ± 7.02 ng/mL
NGP^{r}ETO^{200}	281.37 ± 39.16 ng/mL

Cell line	IC$_{50}$ gemcitabine
NGP	0.045 ± 0.023 ng/mL
NGP^{r}GEMCI^{20}	573.9 ± 162.2 ng/mL
Cell line	IC$_{50}$ irinotecan
NGP	229.89 ± 34.82 ng/mL
NGP^{r}IRINO^{1000}	2520 ± 367 ng/mL
Cell line	IC$_{50}$ melphalan
NGP	430.6 ± 82.5 ng/mL
NGP^{r}MEL^{2000}	2557 ± 308 ng/mL
Cell line	IC$_{50}$ oxaliplatin
NGP	524.1 ± 88.3 ng/mL
NGP^{r}OXALI^{4000}	5136 ± 781 ng/mL
Cell line	IC$_{50}$ paclitaxel
NGP	0.41± 0.08 ng/mL
NGP^{r}PCL^{20}	37.83 ± 5.26 ng/mL
Cell line	IC$_{50}$ topotecan
NGP	1.82 ± 0.30 ng/mL
NGP^{r}TOPO^{20}	1022 ± 237 ng/mL
Cell line	IC$_{50}$ vincristine
NGP	47.6 ± 3.9 ng/mL
NGP^{r}VCR^{100}	187.6 ± 87.7 ng/mL
Cell line	IC$_{50}$ vinblastine
NGP	0.07 ± 0.02 ng/mL
NGP^{r}VINB^{20}	20.81 ± 4.26 ng/mL
Cell line	IC$_{50}$ vinorelbine
NGP	0.43 ± 0.07 ng/mL
NGP^{r}VINOR^{20}	28.2 ± 3.7 ng/mL
Cell line	IC$_{50}$ carboplatin
NLF	1533 ± 196 ng/mL
NLF^{r}CARBO^{5000}	7001 ± 568 ng/mL
Cell line	IC$_{50}$ cisplatin
NLF	228.3 ± 18.5 ng/mL
NLF^{r}CDDP^{1000}	2204 ± 281 ng/mL
Cell line	IC$_{50}$ dacarbazine
NLF	13.75 ± 1.60 µg/mL
NLF^{r}DACARB^{20}	39.26 ± 4.74 µg/mL

Cell line	IC$_{50}$ docetaxel
NLF	0.57 ± 0.06 ng/mL
NLF^{r}DOCE^{20}	69.77 ± 8.07 ng/mL
Cell line	IC$_{50}$ doxorubicin
NLF	10.74 ± 1.03 ng/mL
NLF^{r}DOX^{40}	83.91 ± 16.83 ng/mL
Cell line	IC$_{50}$ etoposide
NLF	47.90 ± 5.16 ng/mL
NLF^{r}ETO^{100}	210.81 ± 2.35 ng/mL
Cell line	IC$_{50}$ gemcitabine
NLF	0.73 ± 0.05 ng/mL
NLF^{r}GEMCI^{20}	76.38 ± 9.48 ng/mL
Cell line	IC$_{50}$ irinotecan
NLF	149.3 ± 19.5 ng/mL
NLF^{r}IRINO^{1000}	2861 ± 331 ng/mL
Cell line	IC$_{50}$ melphalan
NLF	259.5 ± 20.8 ng/mL
NLF^{r}MEL^{3000}	1260 ± 149 ng/mL
Cell line	IC$_{50}$ oxaliplatin
NLF	3296 ± 399 ng/mL
NLF^{r}OXALI^{4000}	11862 ± 982 ng/mL
Cell line	IC$_{50}$ paclitaxel
NLF	0.73 ± 0.11 ng/mL
NLF^{r}PCL^{20}	27.60 ± 3.42 ng/mL
Cell line	IC$_{50}$ topotecan
NLF	1.12 ± 0.15 ng/mL
NLF^{r}TOPO^{10}	14.14 ± 1.39 ng/mL
Cell line	IC$_{50}$ vincristine
NLF	0.017 ± 0.006 ng/mL
NLF^{r}VCR^{5}	7.39 ± 0.28 ng/mL
Cell line	IC$_{50}$ vinblastine
NLF	0.27 ± 0.04 ng/mL
NLF^{r}VINB^{10}	18.27 ± 2.43 ng/mL
Cell line	IC$_{50}$ vinorelbine
NLF	0.067 ± 0.008 ng/mL
NLF^{r}VINOR^{10}	4.61 ± 0.31 ng/mL
Cell line	IC$_{50}$ cisplatin

SHEP	188.5 ± 22.6 ng/mL
SHEP^{r}CDDP^{1000}	1624 ± 182 ng/mL
Cell line	IC$_{50}$ doxorubicin
SHEP	7.65 ± 0.91 ng/mL
SHEP^{r}DOX^{100}	153.7 ± 19.2 ng/mL
Cell line	IC$_{50}$ etoposide
SHEP	40.60 ± 5.56 ng/mL
SHEP^{r}ETO^{100}	258.4 ± 36.3 ng/mL
Cell line	IC$_{50}$ vincristine
SHEP	0.15 ± 0.02 ng/mL
SHEP^{r}VCR^{1}	1.17 ± 0.15 ng/mL
Cell line	IC$_{50}$ cisplatin
SH-SY5Y	59 ± 15 ng/mL
SH-SY5Y^{r}CDDP^{200}	350 ± 41 ng/mL
Cell line	IC$_{50}$ doxorubicin
SH-SY5Y	2.13 ± 0.28 ng/mL
SH-SY5Y^{r}DOX^{10}	11.80 ± 1.06 ng/mL
Cell line	IC$_{50}$ vincristine
SH-SY5Y	0.04 ± 0.01 ng/mL
SH-SY5Y^{r}VCR^{10}	15.15 ± 2.02 ng/mL
Cell line	IC$_{50}$ carboplatin
SK-N-AS	572.6 ± 65.8 ng/mL
SK-N-AS^{r}CARBO^{2000}	17211 ± 2403 ng/mL
Cell line	IC$_{50}$ cisplatin
SK-N-AS	60.1 ± 8.6 ng/mL
SK-N-AS^{r}CDDP^{500}	2877 ± 331 ng/mL
Cell line	IC$_{50}$ dacarbazine
SK-N-AS	9.47 ± 1.13 µg/mL
SK-N-AS^{r}DACARB^{16}	86.17 ± 11.25 µg/mL
Cell line	IC$_{50}$ docetaxel
SK-N-AS	0.05 ± 0.01 ng/mL
SK-N-AS^{r}DOCE^{10}	24.14 ± 4.07 ng/mL
Cell line	IC$_{50}$ doxorubicin
SK-N-AS	6.32 ± 0.82 ng/mL
SK-N-AS^{r}DOX^{10}	15.52 ± 1.97 ng/mL
Cell line	IC$_{50}$ etoposide
SK-N-AS	191.6 ± 24.9 ng/mL

SK-N-AS^{r}ETO^{200}	851.1 ± 144.3 ng/mL
Cell line	IC$_{50}$ gemcitabine
SK-N-AS	0.24 ± 0.04 ng/mL
SK-N-AS^{r}GEMCI^{10}	11.10 ± 1.34 ng/mL
Cell line	IC$_{50}$ irinotecan
SK-N-AS	52.69 ± 6.13 ng/mL
SK-N-AS^{r}IRINO^{500}	525.3 ± 64.9 ng/mL
Cell line	IC$_{50}$ melphalan
SK-N-AS	547.1 ± 89.2 ng/mL
SK-N-AS^{r}MEL^{1000}	1015 ± 152 ng/mL
Cell line	IC$_{50}$ oxaliplatin
SK-N-AS	657.2 ± 86.3 ng/mL
SK-N-AS^{r}OXALI^{4000}	6939 ± 795 ng/mL
Cell line	IC$_{50}$ paclitaxel
SK-N-AS	1.00 ± 0.17 ng/mL
SK-N-AS^{r}PCL^{20}	84.04 ± 12.7 ng/mL
Cell line	IC$_{50}$ topotecan
SK-N-AS	0.69 ± 0.15 ng/mL
SK-N-AS^{r}TOPO^{20}	28.92 ± 3.24 ng/mL
Cell line	IC$_{50}$ vincristine
SK-N-AS	0.17 ± 0.03 ng/mL
SK-N-AS^{r}VCR^{20}	23.75 ± 2.98 ng/mL
Cell line	IC$_{50}$ vinblastine
SK-N-AS	0.020 ± 0.005 ng/mL
SK-N-AS^{r}VINB^{10}	35.03 ± 7.51 ng/mL
Cell line	IC$_{50}$ vinorelbine
SK-N-AS	0.04 ± 0.01 ng/mL
SK-N-AS^{r}VINOR^{20}	16.55 ± 4.85 ng/mL
Cell line	IC$_{50}$ cisplatin
UKF-NB-1	181.9 ± 15.4 ng/mL
UKF-NB-1^{r}CDDP^{1000}	1562 ± 179 ng/mL
Cell line	IC$_{50}$ vincristine
UKF-NB-1	5.09 ± 0.42 ng/mL
UKF-NB-1^{r}VCR^{10}	24.36 ± 2.83 ng/mL
Cell line	IC$_{50}$ carboplatin
UKF-NB-2	919.66 ± 17.83 ng/mL
UKF-NB-2^{r}CARBO^{2000}	8191 ± 126 ng/mL

Cell line	IC$_{50}$ cisplatin
UKF-NB-2	149 ± 18 ng/mL
UKF-NB-2^{r}CDDP^{1000}	2586 ± 207 ng/mL
Cell line	IC$_{50}$ dacarbazine
UKF-NB-2	1.05 ± 0.18 µg/mL
UKF-NB-2^{r}DACARB^{4}	38.86 ± 5.88 µg/mL
Cell line	IC$_{50}$ docetaxel
UKF-NB-2	2.20 ± 0.11 ng/mL
UKF-NB-2^{r}DOCE^{10}	32.91 ± 1.47 ng/mL
Cell line	IC$_{50}$ doxorubicin
UKF-NB-2	7.36 ± 0.80 ng/mL
UKF-NB-2^{r}DOX^{20}	351 ± 40 ng/mL
Cell line	IC$_{50}$ etoposide
UKF-NB-2	44.72 ± 7.41 ng/mL
UKF-NB-2^{r}ETO^{400}	1112 ± 73 ng/mL
Cell line	IC$_{50}$ irinotecan
UKF-NB-2	232 ± 44 ng/mL
UKF-NB-2^{r}IRINO^{400}	2730 ± 366 ng/mL
Cell line	IC$_{50}$ melphalan
UKF-NB-2	299 ± 56 ng/mL
UKF-NB-2^{r}MEL^{600}	8695 ± 856 ng/mL
Cell line	IC$_{50}$ nutlin-3
UKF-NB-2	2.05 ± 0.33 µM
UKF-NB-2^{r}Nutlin^{10µM}	21.03 ± 1.77 µM
Cell line	IC$_{50}$ oxaliplatin
UKF-NB-2	329.92 ± 16.71 ng/mL
UKF-NB-2^{r}OXALI^{600}	1187 ± 66 ng/mL
Cell line	IC$_{50}$ paclitaxel
UKFNB-2	4.03 ± 0.15 ng/mL
UKF-NB-2^{r}PCL^{20}	182.41 ± 16.90 ng/mL
Cell line	IC$_{50}$ topotecan
UKF-NB-2	1.63 ± 0.06 ng/mL
UKF-NB-2^{r}TOPO^{10}	40.71 ± 6.18 ng/mL
Cell line	IC$_{50}$ vincristine
UKF-NB-2	0.27 ± 0.04 ng/mL
UKF-NB-2^{r}VCR^{10}	78.01 ± 10.55 ng/mL

Cell line	IC_{50} vinblastine
UKF-NB-2	0.81 ± 0.11 ng/mL
UKF-NB-2^{r}VINB^{10}	40.10 ± 2.78 ng/mL
Cell line	IC_{50} vinorelbine
UKF-NB-2	2.50 ± 0.09 ng/mL
UKF-NB-2^{r}VINOR^{10}	45.45 ± 1.08 ng/mL
Cell line	IC_{50} bortezomib
UKF-NB-3	3.22 ± 0.39 nM
UKF-NB-3^{r}BORTE^{20}	48.91 ± 6.31 nM
Cell line	IC_{50} cabazitaxel
UKF-NB-3	0.58 ± 0.03 ng/mL
UKF-NB-3^{r}CABAZI^{2}	3.73 ± 0.45 ng/mL
Cell line	IC_{50} carboplatin
UKF-NB-3	539.73 ± 89.40 ng/mL
UKF-NB-3^{r}CARBO^{2000}	8600 ± 559 ng/mL
Cell line	IC_{50} cisplatin
UKF-NB-3	60.14 ± 7.92 ng/mL
UKF-NB-3^{r}CDDP^{1000}	2784 ± 99 ng/mL
Cell line	IC_{50} dacarbazine
UKF-NB-3	3.60 ± 0.42 µg/mL
UKF-NB-3^{r}DACARB^{8}	85.83 ± 10.11 µg/mL
Cell line	IC_{50} docetaxel
UKF-NB-3	0.71 ± 0.21 ng/mL
UKF-NB-^{r}DOCE^{20}	34.89 ± 7.69 ng/mL
Cell line	IC_{50} doxorubicin
UKF-NB-3	7.16 ± 2.35 ng/mL
UKF-NB-^{r}DOX^{20}	67.83 ± 14.73 ng/mL
Cell line	IC_{50} etoposide
UKF-NB-3	27.14 ± 3.05 ng/mL
UKF-NB-3^{r}ETO^{200}	140 ± 17 ng/mL
Cell line	IC_{50} gemcitabine
UKF-NB-3	0.89 ± 0.17 ng/mL
UKF-NB-3^{r}GEMCI^{10}	44.50 ± 7.18 ng/mL
Cell line	IC_{50} irinotecan
UKF-NB-3	23.50 ± 2.72 ng/mL
UKF-NB-3^{r}IRINO^{800}	1781 ± 163 ng/mL
Cell line	IC_{50} 2-methoxyestradiol

UKF-NB-3	143.2 ± 16.7 nM
UKF-NB-3^{r}ME^{1000}	1681 ± 178 nM
Cell line	IC$_{50}$ melphalan
UKF-NB-3	83.74 ± 11.06 ng/mL
UKF-NB-3^{r}MEL^{400}	485 ± 58 ng/mL
Cell line	IC$_{50}$ nutlin-3
UKF-NB-3	0.68 ± 0.09 µM
UKF-NB-3^{r}Nutlin^{10µM}	23.90 ± 2.53 µM
Cell line	IC$_{50}$ NVP-TAE684
UKF-NB-3	62.3 ± 8.3 nM
UKF-NB-3^{r}NVP-TAE684^{500}	1315 ± 152 nM
Cell line	IC$_{50}$ oxaliplatin
UKF-NB-3	212.4 ± 5.11 ng/mL
UKF-NB-3^{r}OXALI^{2000}	3242 ± 343 ng/mL
Cell line	IC$_{50}$ paclitaxel
UKF-NB-3	1.12 ± 0.15 ng/mL
UKF-NB-3^{r}PCL^{20}	145.65 ± 20.02 ng/mL
Cell line	IC$_{50}$ RITA
UKF-NB-3	0.11 ± 0.04 µM
UKF-NB-3^{r}RITA^{10µM}	13.48 ± 2.35 µM
Cell line	IC$_{50}$ topotecan
UKF-NB-3	2.52 ± 0.25 ng/mL
UKF-NB-3^{r}TOPO^{15}	64.92 ± 18.40 ng/mL
Cell line	IC$_{50}$ vincristine
UKF-NB-3	0.44 ± 0.09 ng/mL
UKF-NB-3^{r}VCR^{10}	73.32 ± 17.36 ng/mL
Cell line	IC$_{50}$ vinblastine
UKF-NB-3	0.20 ± 0.17 ng/mL
UKF-NB-3^{r}VINB^{10}	22.19 ± 5.48 ng/mL
Cell line	IC$_{50}$ vinorelbine
UKF-NB-3	1.03 ± 0.11 ng/mL
UKF-NB-3^{r}VINOR^{20}	116 ± 7 ng/mL
Cell line	IC$_{50}$ YM155
UKF-NB-3	0.41 ± 0.08 nM
UKF-NB-3^{r}YM155^{50nM}	70.80 ± 15.22 nM
Cell line	IC$_{50}$ cisplatin
UKF-NB-4	729 ± 63 ng/mL

UKF-NB-4^{r}CDDP^{1000}	1044 ± 55 ng/mL
Cell line	IC$_{50}$ doxorubicin
UKF-NB-4	95.0 ± 5.1 ng/mL
UKF-NB-4^{r}DOX^{350}	1016 ± 140 ng/mL
Cell line	IC$_{50}$ vincristine
UKF-NB-4	6.89 ± 0.29 ng/mL
UKF-NB-4^{r}VCR^{50}	126.5 ± 4.8 ng/mL
Cell line	IC$_{50}$ carboplatin
UKF-NB-6	927 ± 122 ng/mL
UKF-NB-6^{r}CARBO^{2000}	4560 ± 515 ng/mL
Cell line	IC$_{50}$ cisplatin
UKF-NB-6	340 ± 50 ng/mL
UKF-NB-6^{r}CDDP^{1000}	3543 ± 721 ng/mL
Cell line	IC$_{50}$ dacarbazine
UKF-NB-6	4.38 ± 0.51 µg/mL
UKF-NB-6^{r}DACARB^{8}	35.16 ± 4.02 µg/mL
Cell line	IC$_{50}$ docetaxel
UKF-NB-6	0.26 ± 0.05 ng/mL
UKF-NB-6^{r}DOCE^{10}	12.78 ± 1.52 ng/mL
Cell line	IC$_{50}$ doxorubicin
UKF-NB-6	6.05 ± 0.92 ng/mL
UKF-NB-6^{r}DOX^{20}	241.12 ± 52.81 ng/mL
Cell line	IC$_{50}$ etoposide
UKF-NB-6	117 ± 22 ng/mL
UKF-NB-6^{r}ETO^{200}	760 ± 224 ng/mL
Cell line	IC$_{50}$ gemcitabine
UKF-NB-6	0.74 ± 0.22 ng/mL
UKF-NB-6^{r}GEMCI^{10}	13.77 ± 1.45 ng/mL
Cell line	IC$_{50}$ irinotecan
UKF-NB-6	404.5 ± 38.4 ng/mL
UKF-NB-6^{r}IRINO^{1000}	7769 ± 882 ng/mL
Cell line	IC$_{50}$ melphalan
UKF-NB-6	418 ± 14 ng/mL
UKF-NB-6^{r}MEL^{1000}	1272 ± 22 ng/mL
Cell line	IC$_{50}$ oxaliplatin
UKF-NB-6	688.85 ± 30.62 ng/mL
UKF-NB-6^{r}OXALI^{4000}	5875 ± 360 ng/mL

Cell line	IC_{50} paclitaxel
UKF-NB-6	1.03 ± 0.09 ng/mL
UKF-NB-6^{r}PCL^{20}	37.91 ± 6.47 ng/mL
Cell line	IC_{50} topotecan
UKF-NB-6	2.50 ± 0.51 ng/mL
UKF-NB-6^{r}TOPO^{20}	27.52 ± 3.07 ng/mL
Cell line	IC_{50} vincristine
UKF-NB-6	1.25 ± 0.32 ng/mL
UKF-NB-6^{r}VCR^{10}	55.03 ± 5.25 ng/mL
Cell line	IC_{50} vinblastine
UKF-NB-6	0.46 ± 0.12 ng/mL
UKF-NB-6^{r}VINB^{10}	16.15 ± 2.08 ng/mL
Cell line	IC_{50} vinorelbine
UKF-NB-6	1.13 ± 0.12 ng/mL
UKF-NB-6^{r}VINOR^{40}	65.57 ± 14.98 ng/mL
Cell line	IC_{50} YM155
UKF-NB-6	0.52 ± 0.16 nM
UKF-NB-6^{r}YM155^{20nM}	123.33 ± 11.57 nM

Suppl. Table S11. Target sequences of the used siRNA pools.

CDKN1A (p21):
- CGACUGUGAUGCGCUAAUG
- CCUAAUCCGCCCACAGGAA
- CGUCAGAACCCAUGCGGCA
- AGACCAGCAUGACAGAUUU

BAX:
GUGCCGGAACUGAUCAGAA
ACAUGUUUUCUGACGGCAA
CUGAGCAGAUCAUGAAGAC
UGGGCUGGAUCCAAGACCA

BBC3 (PUMA):
CGGACGACCUCAACGCACA
CCGAGAUGGAGCCCAAUUA
CCUGGAGGGUCCUGUACAA
GUAGAUACCGGAAUGAAUU

CASP9 (caspase 9):
GAGUCAGGCUCUUCCUUUG
CGGUGAAAGGGAUUUAUAA
UCAUAGAUCUGGAGACUCG
GCAGAAAGACCAUGGGUUU

Non targeting
UGGUUUACAUGUCGACUAA
UGGUUUACAUGUUGUGUGA
UGGUUUACAUGUUUUCUGA
UGGUUUACAUGUUUUCCUA
